# Application of proteomics to determine the mechanism of ozone on sweet cherries (*Prunus avium* L.) by time-series analysis

**DOI:** 10.3389/fpls.2023.1065465

**Published:** 2023-02-09

**Authors:** Yuehan Zhao, Zhaohua Hou, Na Zhang, Haipeng Ji, Chenghu Dong, Jinze Yu, Xueling Chen, Cunkun Chen, Honglian Guo

**Affiliations:** ^1^ College of Food Science and Engineering, Tianjin University of Science and Technology, Tianjin, China; ^2^ Institute of Agricultural Products Preservation and Processing Technology (National Engineering Technology Research Center for Preservation of Agriculture Product), Tianjin Academy of Agricultural Sciences, Key Laboratory of Postharvest Physiology and Storage of Agricultural Products, Ministry of Agriculture of the People’s Republic of China, Tianjin Key Laboratory of Postharvest Physiology and Storage of Agricultural Products, Tianjin, China; ^3^ College of Food Science and Engineering, Qilu University of Technology (Shandong Academy of Sciences), Ji’nan, China; ^4^ Key Laboratory of Cold Chain Logistics Technology for Agro-product, Ministry of Agriculture and Rural Affairs, Ministry of Agriculture and Rural Affairs, Institute of Agro-Products Processing and Nuclear agricultural Technology, Hubei Academy of Agricultural Sciences, Wuhan, China

**Keywords:** sweet cherry (*Prunus avium* L.), ozone, label-free quantification proteomics, preservation, mfuzz

## Abstract

This research investigated the mechanism of ozone treatment on sweet cherry (*Prunus avium* L.) by Lable-free quantification proteomics and physiological traits. The results showed that 4557 master proteins were identified in all the samples, and 3149 proteins were common to all groups. Mfuzz analyses revealed 3149 candidate proteins. KEGG annotation and enrichment analysis showed proteins related to carbohydrate and energy metabolism, protein, amino acids, and nucleotide sugar biosynthesis and degradation, and fruit parameters were characterized and quantified. The conclusions were supported by the fact that the qRT-PCR results agreed with the proteomics results. For the first time, this study reveals the mechanism of cherry in response to ozone treatment at a proteome level.

## Introduction

Sweet cherry (*Prunus avium* L.) is an economically important horticultural crop cultivated in north China, which is thought to be a non-climacteric fruit. Sweet cherries are a significant and valuable fruit that are a fantastic source of many phytochemicals with health-promoting qualities (anthocyanins, vitamins, phenolics etc.), and are widely adored by consumers (Zhang et al., 2007; Calle et al., 2021; Clayton-Cuch et al., 2021; Gutierrez et al., 2021). However, several physiological or environmental conditions will have a significant impact on cherry fruits during storage, which will reduce their post-harvest quality and market value. Postharvest loss is a serious issue that affects the entire world, each year, more than one-third of fruits and vegetables are lost in the field and the subsequent supply chain (Romanazzi et al., 2016).

Because it safely and spontaneously breaks down into oxygen and free radicals quickly without leaving any lasting effects, ozone has gained acceptance as a food sanitizer, particularly in organic farming (Monaco et al., 2016). In 2001, ozone was approved by the FDA for the treatment of fresh fruit (FDA, 2001). A thorough study is currently being conducted on the ozonation method, which increases the shelf life of stored raw fruits and vegetables. For instance, numerous attempts have been made to use ozone therapy to lengthen the shelf life of carrot, lettuce, peach and berries (Tabakoglu and Karaca, 2018). Many papers have proved that gaseous ozone is also use to reduce formation of volatile chemicals, such as propene or acetylene, which induce unfavorable early fruit ripening. Initial research has mainly concentrated on the study of ozone to destroy hazardous microbes. Studies have shown that changes in the content of antioxidant components, such as flavonoids and other phenolic compounds, are positively impacted by gaseous ozone. Ozone can, however, damage nutrients, change the appearance of food, and impair food flavor when used improperly (Sachadyn-Kró et al., 2016). However, it is unknown how this potent oxidant may affect the food’s quality and safety for consumption.

The development of omics techniques has made it feasible to obtain understanding of the intricate processes that take place during cherry storage at the protein level. Proteome profiling techniques that reveal changes in protein content during fruits storage are currently uncommon and tend to concentrate mature or germination-stage seeds. In the current study, the dynamic change of proteins in cherry-treated ozone during cold stress stage was investigated using a label-free proteomics and bioinformatics analysis. This study, as far as we are aware, was the first to look at the molecular mechanism of ozone in cherry preservation. The findings provide vital information for cherry preservation as well as information on cherry molecular responses to ozone.

## Materials and methods

### Sample collection and treatment

**Figure 1:**
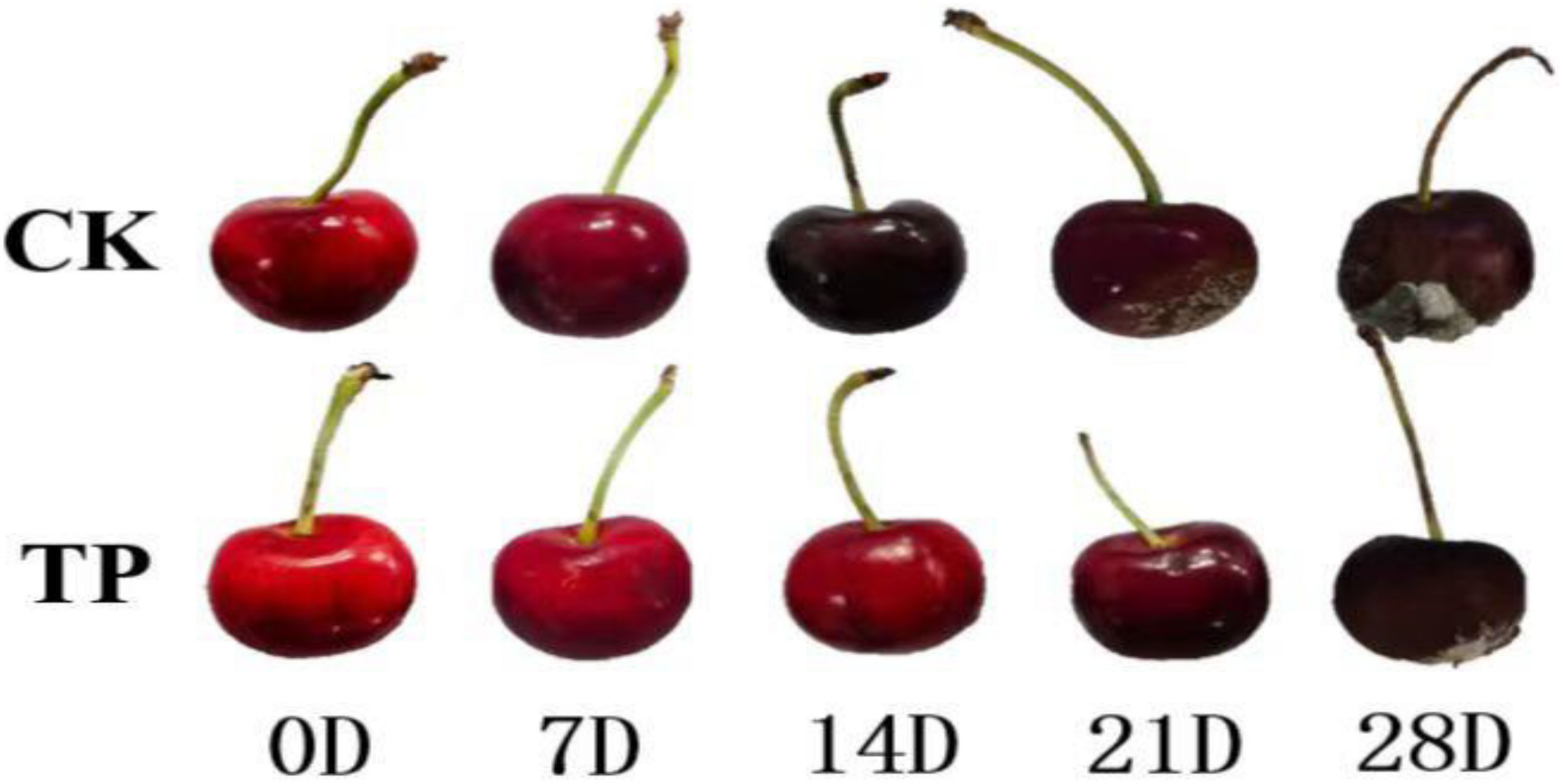
The visual appearance of different treatment cherry fruit stored for 28 days at 0°C.

Fresh “red lantern” sweet cherries that were undamaged, and commercially mature were acquired on June 1, 2020, in Ninghe District of Tianjin, China, and delivered to the National Engineering Technology Research Center for Agricultural Products Preservation (Tianjin). The samples were separated into two groups (ozone treatment group, TP and control check group, CK) and stored for 35 days at a temperature of 0 ± 0.5°C with a relative humidity of 90 ± 5%. One ozone treatment group (TP) was housed in 18 boxes with 1 kilogram of cherries each, each inside a huge tent. The cherry ozone treatment approach is based on the work of Gao et al. (2020). The ozone sensor (MIC-03, Shenzhen) is fixed in the huge tent with two fans of 0.4 m·s^-1^ generating a breeze of 0.2 m·s^-1^. Every 7 days, the TP sample was fumigated with 3 ppm ozone for two hours. Only the other control check group (CK) was boxed. At 0, 7, 14, 21, 28 and 35 days, physiological indicators and samples were collected for storage (**Figure 1**). After being instantly frozen in liquid nitrogen, the cherry was kept at −80°C until protein and RNA extraction.

### Physical and physiological analysis

#### Soluble solids, titratabole acidity, and firmness

Cherry samples were crushed in a mortar and passed through gauze to determine of the soluble solid content. Utilizing a PLA-1 digital hand-held pocket refractometer, the produced juice was examined (Atago Co., Ltd., Tokyo, Japan). Three times were used to repeat each measurement. The results are given as percentages (%) (Yang SB et al., 2021). Titratable acidity content was determined by titration. Three times were used to repeat each measurement. The results are shown as percentages (%) (Yang SB et al., 2021). Randomly select 10 sweet cherries, and the firmness of the sample were measured with TA.XT.Plus physical property analyzer along the two ends of the sweet cherry equator. The measurement parameters are: probe P/2 (Φ2 mm), puncture preparation speed is 2.00 mm/s, puncture speed is 2.00 mm/s, and puncture depth is 5mm. The result is indicated by “N” (Hu et al., 2020).

#### Measurement of total flavonoids, anthocycadins, total phenol, and ascorbic acid (Vc) content

The method of Zhao et al. (2021) was slightly modified to determine the total flavonoids, anthocycadins, total phenol, and ascorbic acid (Vc) content. The total phenol results were expressed as gram gallic acid equivalents per kilogram fresh weight (mg GA kg^-1^). Ascorbic acid results were expressed as milligram of ascorbic acid for kilograms (mg 100g^-1^ FW).

### Measurement of key antioxidant enzyme activity

Activities of CAT, APX, POD, and SOD were determined as described by Porcher et al. (2020) and Mekontso et al. (2021), with slight alterations. All enzyme activity units were reported as U g^−1^.

### Protein extraction, digestion and analysis

Protein was extracted from sweet cherry using the method that we previously established in studies that have been published (Huang and Hou, 2021). The powdered cherry samples were treated with trichloroacetic acid (TCA)-acetone extraction, and the crude proteins were lysated by urea extraction buffer (8 M urea, 0.1 M Tris-HCl, pH 8.5) and sonicated for 5 min (pulse 2 s on, 3 s off, 50% amplitude) in ice-water bath (BILON-650Y Ultrasonic cell pulverizer). Then, the protein is enzymatically hydrolyzed by FASP method, briefly, total protein (100μg) was loaded into the ultrafiltration centrifuge tubes (10 kD Microcon, Millipore), which then were centrifuged at 14,000×*g* for 15 min. After centrifugation, concentrates were reduced using 100 μL of 100 mM DTT-50mM NH_4_HCO_3_ (50°C, 30 min). Then, 100μL of 100 mM iodoacetamide (IAA) in urea extraction buffer was added, and incubated for 40 min in darkness. The filters were washed with 100μL of urea extraction buffer three times, and followed by 100μL of 25 mM NH_4_HCO_3_ buffer. The concentrate was then digested at 37°C for 18 hours with 200μL of trypsin (Promega), and the resulting peptides were collected as the filtrate.

The peptides were examined using an online Ultimate 3000 nanoflow liquid chromatography tandem Q-Extractive HF mass spectrometer (Thermo Scientific, Waltham, MA, USA). Briefly, the peptide mixture was loaded onto a reversed-phase trap column (Thermo Scientific Acclaim PepMap 100, 100μm×2cm, nanoViper C_18_) connected to a C18 reversed-phase analytical column (Thermo Scientific Acclaim PepMap 100, 15 cm long, 75 μm inner diameter, 3μm resin). MS data were obtained using a top 20 data-dependent acquirement (DDA) dynamically choosing the most abundant precursor ions from the survey scan (*m/z*150~2000) for higher-energy collisional dissociation (HCD) fragmentation. The following settings were made to the instrument: automatic gain control target was 3e^6^; dynamic exclusion duration, 30 s; resolution for survey scans, 120,000 at *m/z* 200; resolution for HCD spectra, 30,000 at *m/z* 200, and isolation width was *m/z* 2. The normalized collision energy was 27, and the underfill ratio was 0.1%. Each sample was analyzed in three technical triplicates (Huang and Hou, 2021).

### Protein identification

Proteome Discoverer software (ver. 2.2, Thermo Scientific, Bremen, Germany) was utilized to evaluate the raw data, searches against UniProtKB/Swiss-Prot cherry database (release 2020_12, 35660 total entries, downloaded 12/26/20) were performed. Search parameters were: precursor error tolerance 10 ppm, fragment ion tolerance 0.5 Da, trypsin full specificity, maximum number of missed cleavages 2. Methionine oxidation and protein N-term acetylation were set as variable modifications and cysteine carbamidomethylation was designated as fixed modification. False discovery rate (FDR) <0.01. Razor and unique peptides were used for protein quantification (Huang and Hou, 2021).

### Database searching and protein quantification

To assess the quality of the proteomic data, the coefficient of variation (CV) distribution for each sample was less than 10%. Unsupervised principal component analysis (PCA) was accomplished using R package “prcomp” within R language (www.r-project.org) and the data were scaled to zero-mean and unit variance before PCA (Li QQ et al., 2022; Li Y et al., 2022). Differential expression between TP/CK groups was analyzed with the limma R package (1.10.1). The resulting P values were adjusted according to the Benjamini and Hochberg approach for controlling the false discovery rate. Differentially expressed proteins were those determined by R package limma with an adjusted P-value < 0.05, |log2 (fold change)| > 1, false discovery rate < 0.01. (Yu et al., 2019).

### Soft clustering analysis

Soft clustering can assign a gene to many clusters using the fuzzy c-means algorithm with time-course data on the gene expression (Kumar and Futschik, 2007). To analyze the expression trend of proteins with time, R software packages “Mfuzz” was used to soft clustering, which generates the membership values of proteins in a cluster to reflect the degree of a gene’s association with a cluster (Yuan and Cai, 2017). The similarity of protein expression vectors to each other can be determined by membership value (Yang CH et al., 2021).

### Bioinformatics analysis

The KEGG (Kyoto Encyclopedia of Genes and Genomes) enrichment analysis is useful to map unigenes onto known signaling pathways (Yu et al., 2019). The “clusterProfiler” package in R was used to conduct the functional analysis for DEPs in CK and TP groups, respectively. A hypergeometric distribution test was used to obtain the P-value of enriched pathways, Subsequently, the P-value was revised using BH (Benjamini and Hochberg) approach, and the adjusted P-value < 0.05 was served as the cut-off criterion (Yang CH et al., 2021).

### Quantitative real-time PCR analysis

Eight randomly selected proteins were used to analyze the transcription level using qRT-PCR with GAPDH (GenBank No. LOC107418185) as the reference gene (Yang CH et al., 2021). Total RNA extraction, primer design, and PCR detection were described in the literature (Yang CH et al., 2021; Dahn et al., 2021). The information of the primers was shown in **Table 1**.

**Table 1:** Sequence of forward and reverse qRT-PCR primers by gene (feature ID).

Feature ID	Forward (5’-3’)	Reverse (5’-3’)
*LOC110759612*	AGTCGCTCAATCTCTCATCG	TACCCATTAAGCCTTGACAT
*LOC110750545*	GAAACACCTCGTAAACCCTA	TTTGAGGGGAATGAATCTGG
*LOC110753532*	TTTTCTTGAGACAGAGAGCC	TATCACTGCCATCCAGAGTA
*LOC110770218*	GACACATTAGCAAAAACCCT	TTGTTCATGGAGCAACTACA
*LOC110762082*	TCTTCTATATCGCGAAACCC	AGAATTCATTCAATGCCCCT
*LOC110756609*	AAACAACGTTTGAAGGTTCC	ACAGTAGAAAGAAATGCAAAGG
*LOC110760963*	TCAACCAGGAACTGAAAAG	AAAACACATCATCAATGGCG
*LOC110766523*	ATCGGTCTCTTCTTCTACT	TAGCCAGAGAAGAAGATGA

## Results

### Physical parameters

**Figure 2:**
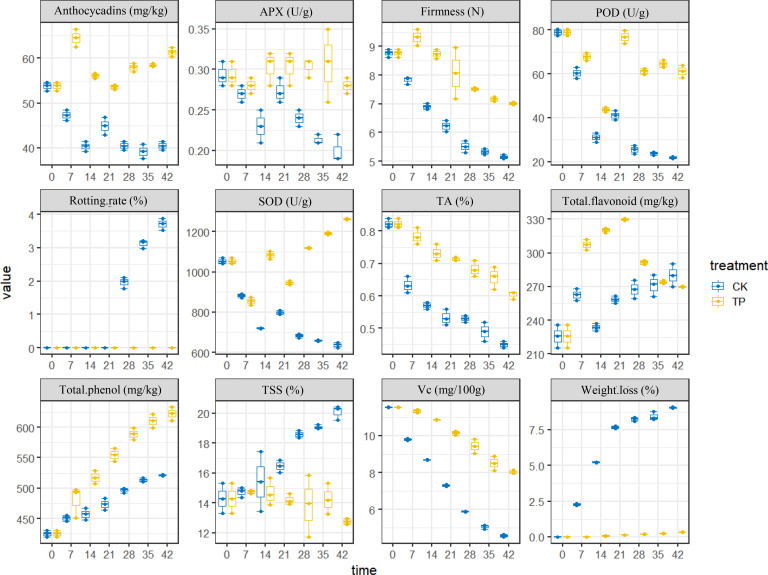
The 12 physiological characteristics of sweet cherry.

The physiological indicators of cherries were detected (**Table S1**). For an intuitive view of the distribution of the different indicators between CK and TP groups, the boxplot was used for analysis. The box plot in **Figure 2** shows the relative levels and changes of the 12 compounds (color, firmness, weight loss, pH, TSS content, and Vc content etc.) in sweet cherry. The level of weight loss, rotting rate and TSS was highest in the CK group and approximately twofold higher than the ozone treatment group. However, the other indicators of SOD, POD, firmness were higher in the ozone treatment, this result is consistent with previous literature reports (Wang et al., 2022). These results suggest a relationship between ozone treatment and traits, ozone can significantly affect the parameters of cherry.

Firmness is the primary external quality factor affecting the marketability. Additional, the firmness of cherry play a crucial role in estimating how long it will preserve. **Figure 2** showed that the firmness of cherry decreased with storage time in all groups, and the CK group was more significant, since it depends on the progress of the conversion of insoluble pectin to soluble pectin or even pectin acid. The firmness of cherry treated ozone with decreased by 26.48%, which is similar to the result reported by Zhang et al. (2021). As a result, the ozone can efficiently retards the softening of sweet cherry. Weight loss and rotting rate increased gradually throughout the storage time. The application of ozone treatment reduced the weight loss within the range of 0 to 0.32% compared with the control (range of 0-9.01%) from 0 to 42 days at 0°C (**Figure 2**). Compared to the control group, the ozone treatment enhanced SOD, POD, and APX activities in sweet cherry. APX and POD activities in treated fruit were increased on 28 days at 0°C, reaching a peak value, respectively. However, SOD activity was higher in treated fruit than that in control during the periods of storage (**Figure 2**).

### Protein profiling from proteomics analysis

**Figure 3:**
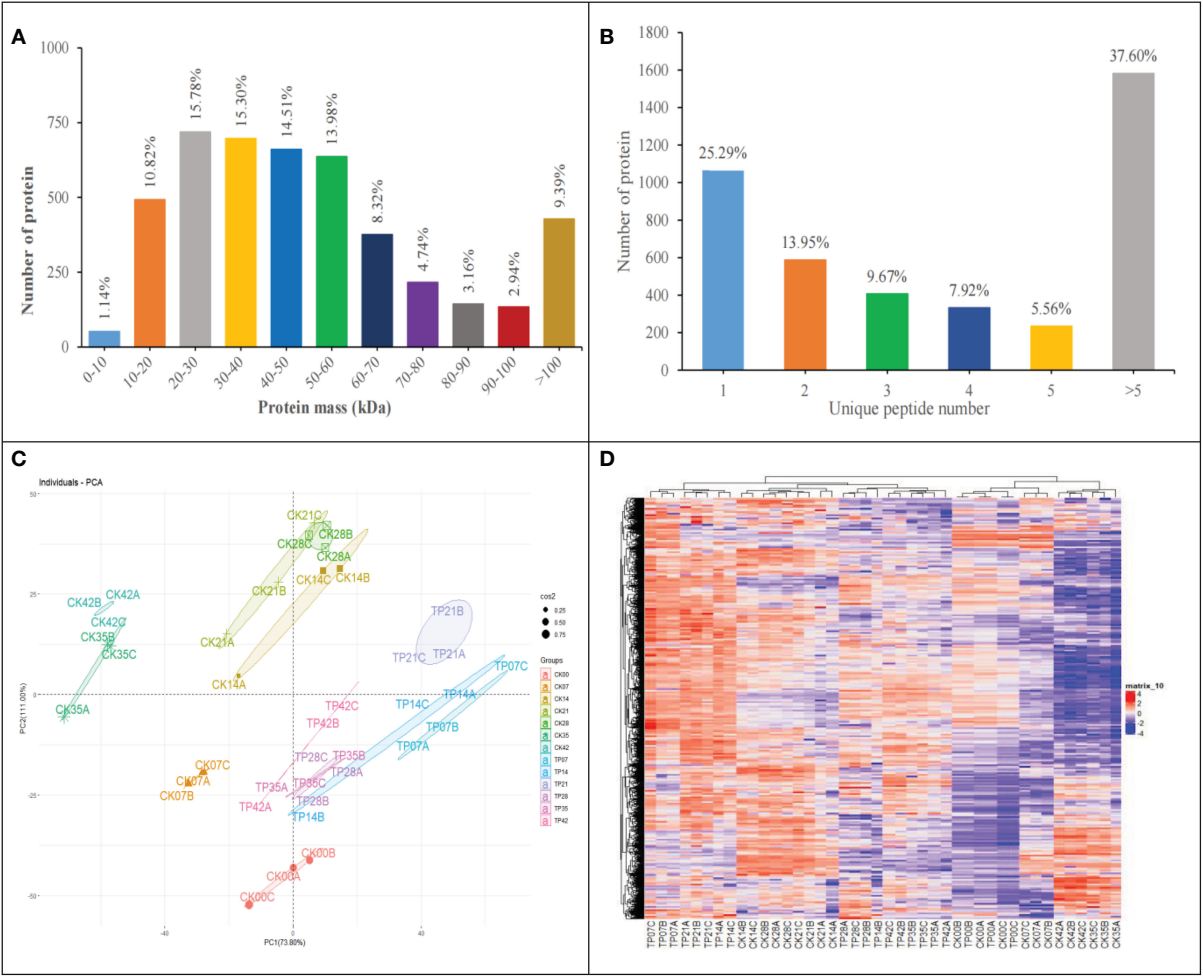
Molecular weight distribution plot of all identified proteins **(A)**. Distribution of proteins containing the different number of identified unique peptides **(B)**. Principal component analysis of protein expression patterns **(C)**. Heatmap of the 4457 identified proteins, different columns represent independent biological replicates **(D)**.

1335877 spectra were acquired using Label-free proteome analysis. After data filtering to eliminate low-scoring spectra, 624157 PSMs identified spectra were matched to 31155 peptides. Finally, total 4557 master proteins were identified with FDR of peptides was less than 0.01, 74.71% proteins were covered with 2 peptides or more (**Figure 3B**).

The number of quantified proteins reached 90.69% of the total identified proteins, accounted for most molecular weight 0-10 kDa (1.14%), 10-20 kDa (10.82%), 20-30 kDa (15.78%), 30-40 kDa (15.30%), 40-50 kDa (14.51%), 50-60 kDa (13.98%), 60-70 kDa (8.32%), 70-80 kDa (4.74%), 80-90 kDa (3.16%) and 90-100 kDa (2.94%) (**Figure 3A**).

**Figures 3C, D** display PCA and HCA based on the 4557 master proteins. As indicated in **Figure 3C**, three replicates of each treatment were flocked together and each sample group was separated well at 13 sampling time points, demonstrating the reliabiliy of the sample collection and analysis; PCA revealed that the first principal component (PC1) reached 73.80% of the variance, and the second one (PC2) reached 11.00% of the variance, the contribution rate of PC1 and PC2 exceeds 80%. These results showed that the cherry fruits were affected significantly in the overall proteome dynamics, providing a suitable experimental dataset. HCA (**Figure 3D**) showed the association among samples (pearson correlation coefficient) according to the overall proteome profile, indicating that three replicates in the same stages had a high correlation (**Figure 3D**).

### Expression cluster pattern of DEPs based on time course

As seen in **Figure S1** and **Table S2**, 3149 proteins were common to the 13 samples. 3149 proteins used for Mfuzz analysis, which were selected by maximum {log2(Abundance + 1)} > 1 and the standard deviation of log2(Abundance + 1) > 1.

**Figure 4:**
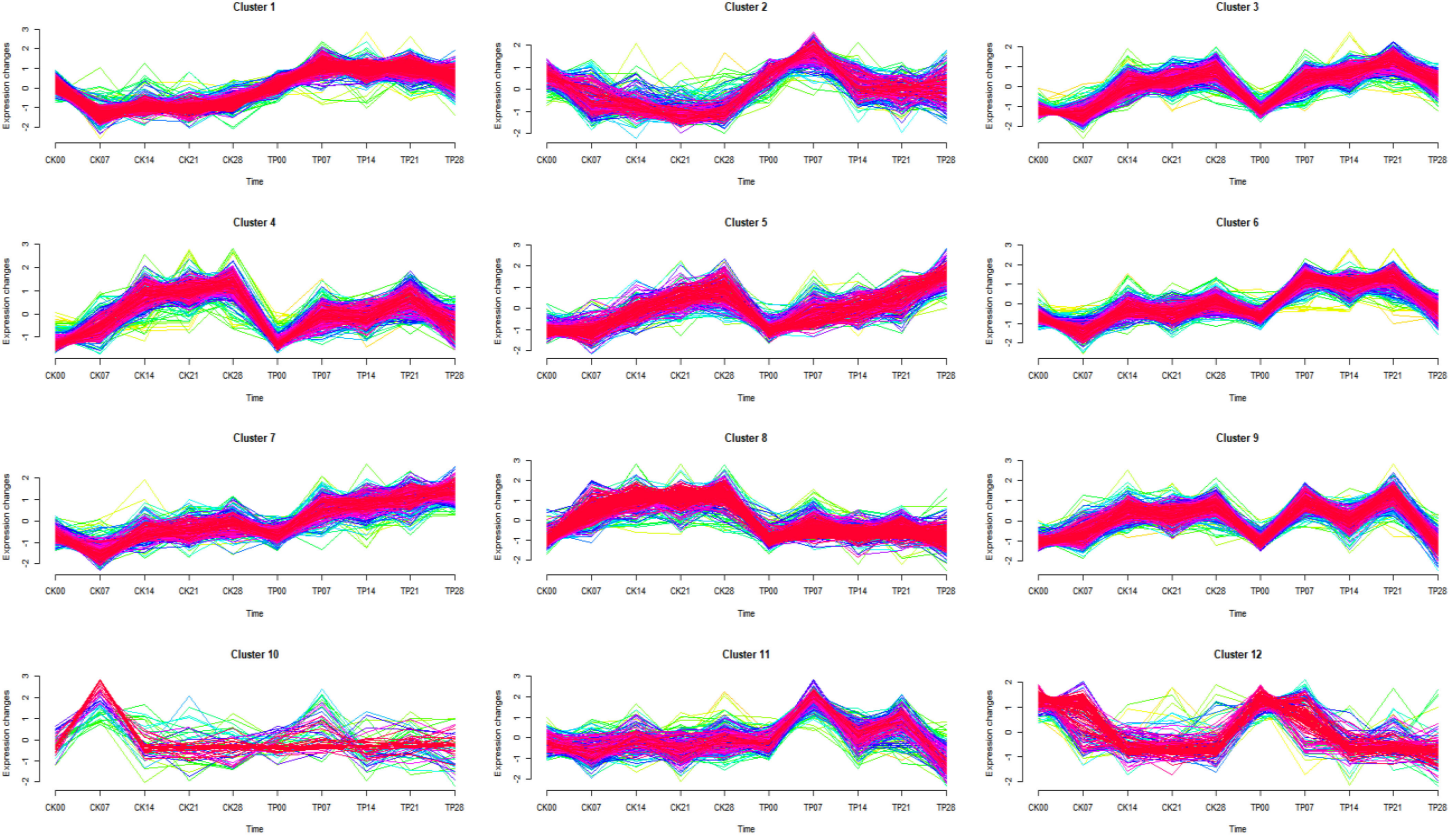
Results of the Mfuzz clustering of 3149 differentially expressed proteins identified by Label-free proteomics based on their expression patterns.

Soft clustering analysis (Mfuzz) was used to observe the trend of differentially expressed proteins with time series, and 12 protein clusters showing an appropriate separation were found (**Figure 4**) (Meng et al., 2018). Mfuzz results showed the 12 proteins clusters, cluster 1 (293 proteins), cluster 2 (407 proteins), cluster 3 (154 proteins), cluster 4 (252 proteins), cluster 5 (284 proteins), cluster 6 (331 proteins), cluster 7 (183 proteins), cluster 8 (294 proteins), cluster 9 (264 proteins), cluster 10 (81 proteins), cluster 11 (455 proteins) and cluster 12 (151 proteins).

Different clusters are merged into 4 groups, according to their trends (**Table 2**). The group 1 from cluster 1, cluster 5, and cluster 7 gradually continuous increased with a time-dependent manner, and the protein content in CK was lower than that in TP at the later stage. The group 2 from cluster 2, and cluster 12 gradually continuous decrease decreased with a time-dependent manner, and the protein content in CK was lower than that in TP at the later stage. The group 3 from cluster 3, cluster 4, cluster 6, cluster 9, and cluster 11 gradually increase first then decreased with a time-dependent manner, and the protein content in CK was lower than that in TP at the later stage. The group 4 from cluster 8 and cluster 10 stable gradually with a time-dependent manner, and protein abundance in CK is higher than that in TP at the later stage. The proteins in the four groups were subjected to KEGG annotation and enrichment analysis (**Figure S2** and **Table 2**). As the biochemcial parameters of cherries gradually decreases with time during storage, 1456 proteins from group 3 with gradually down-regulation in serial passages were selected for further study.

**Table 2:** subcluster of proteins of differential expression protein based on Mfuzz cluster analysis.

Group	clusters	description	Numbers of Proteins	KEGG pathway
Group1	1,5,7	Continuous increase	760	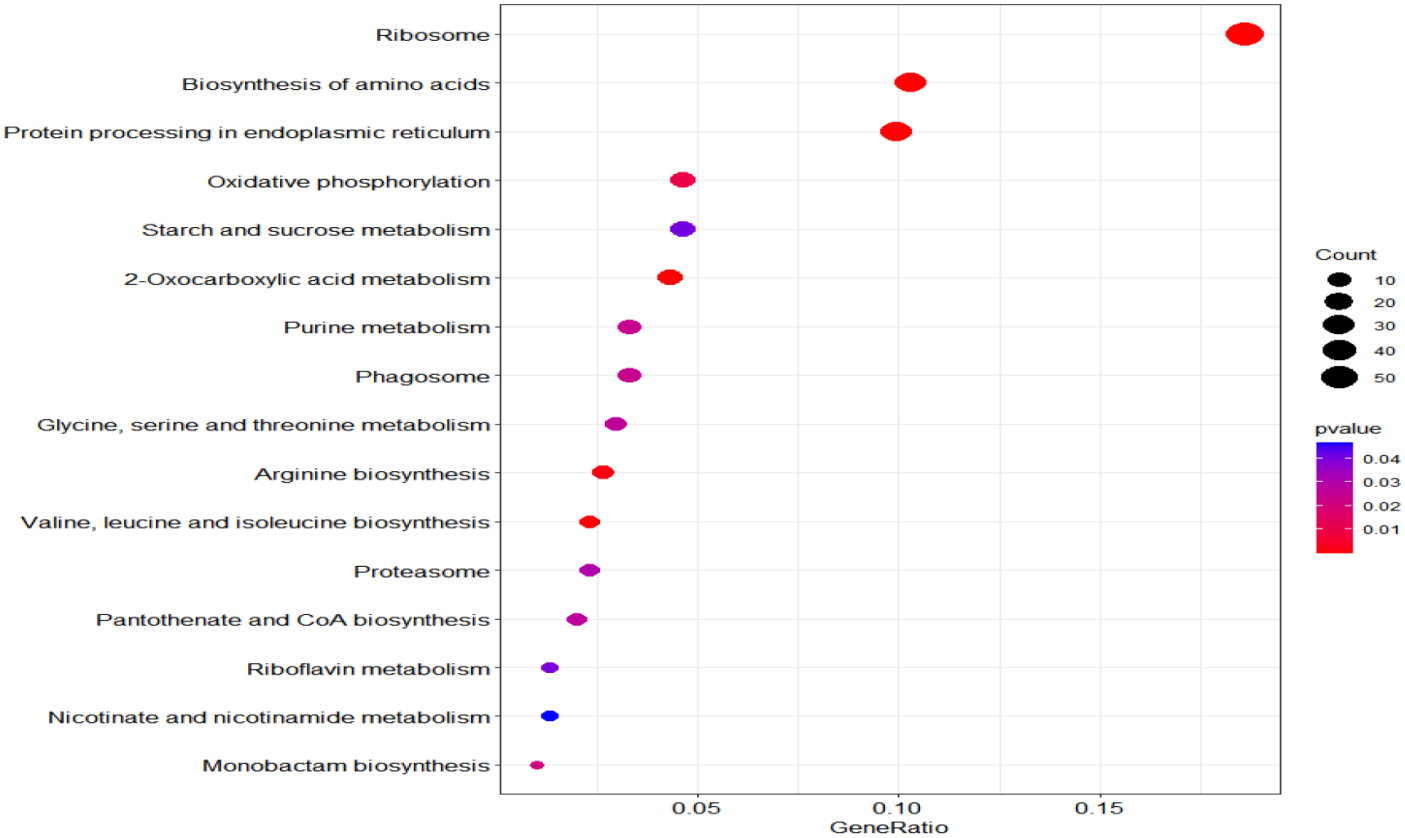
Group2	2,12	Continuous decrease	558	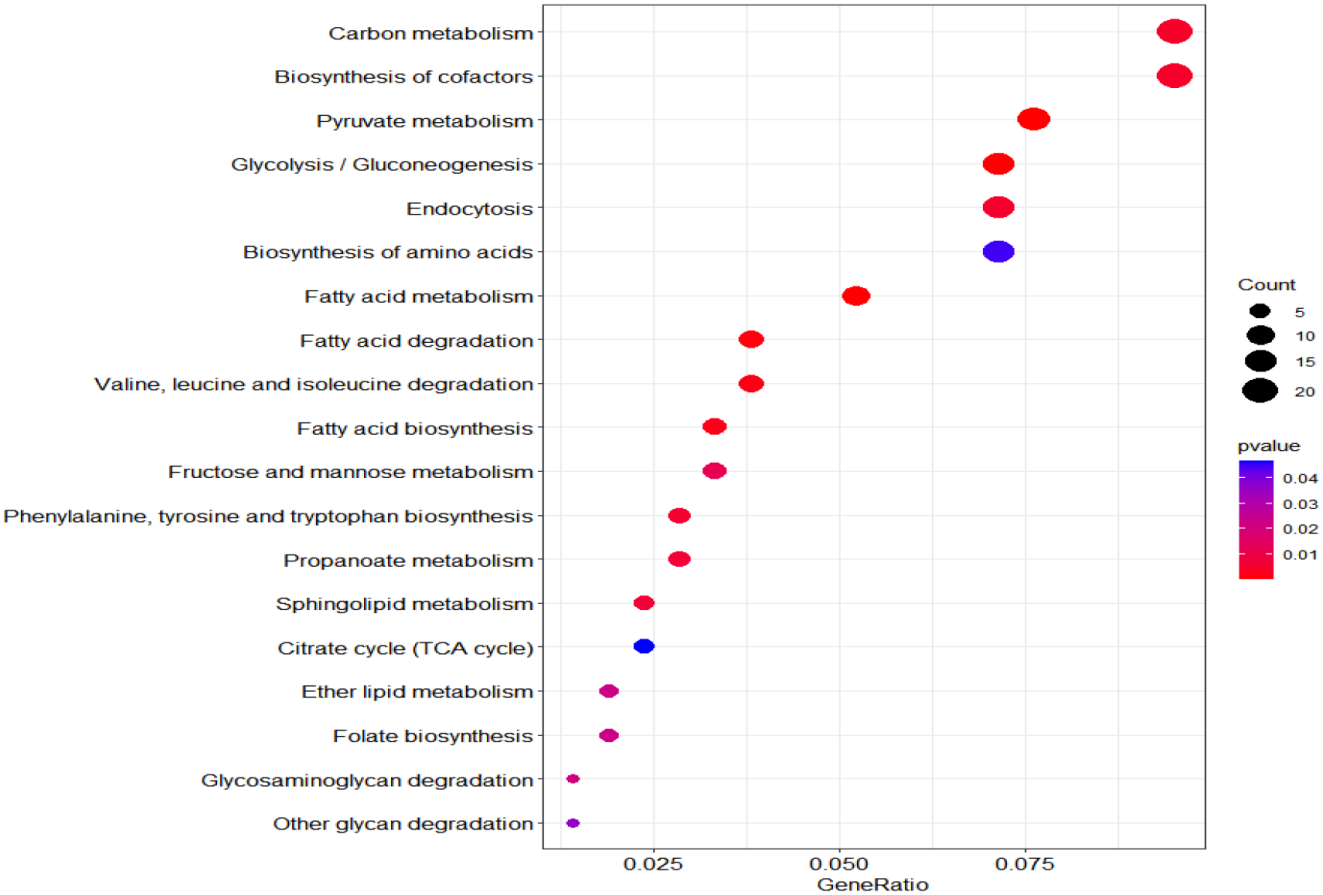
Group3	3,4,6,9,11	Increase first then decrease	1456	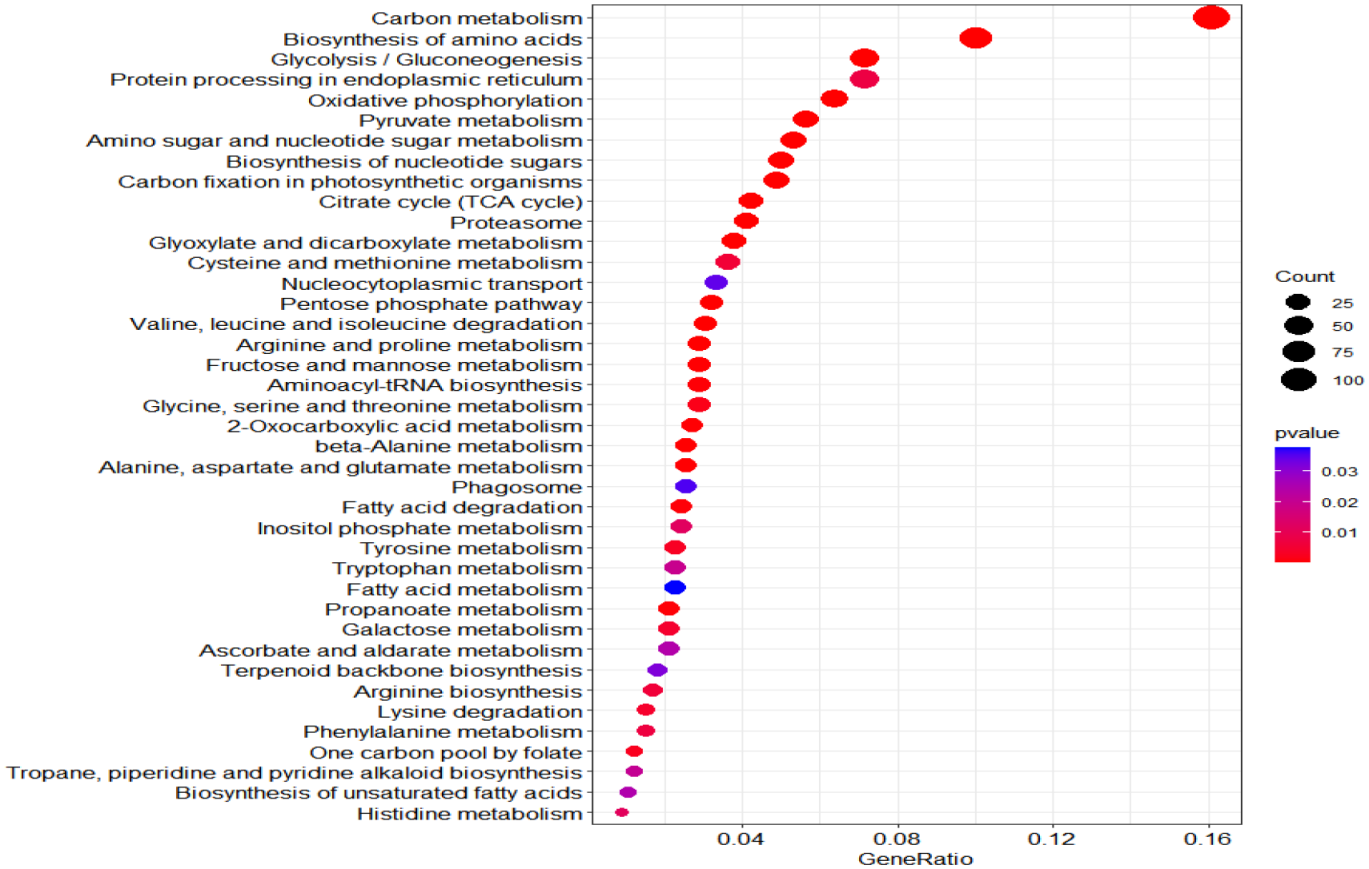
Group4	8,10	Protein abundance in CK is higher than that in TP	375	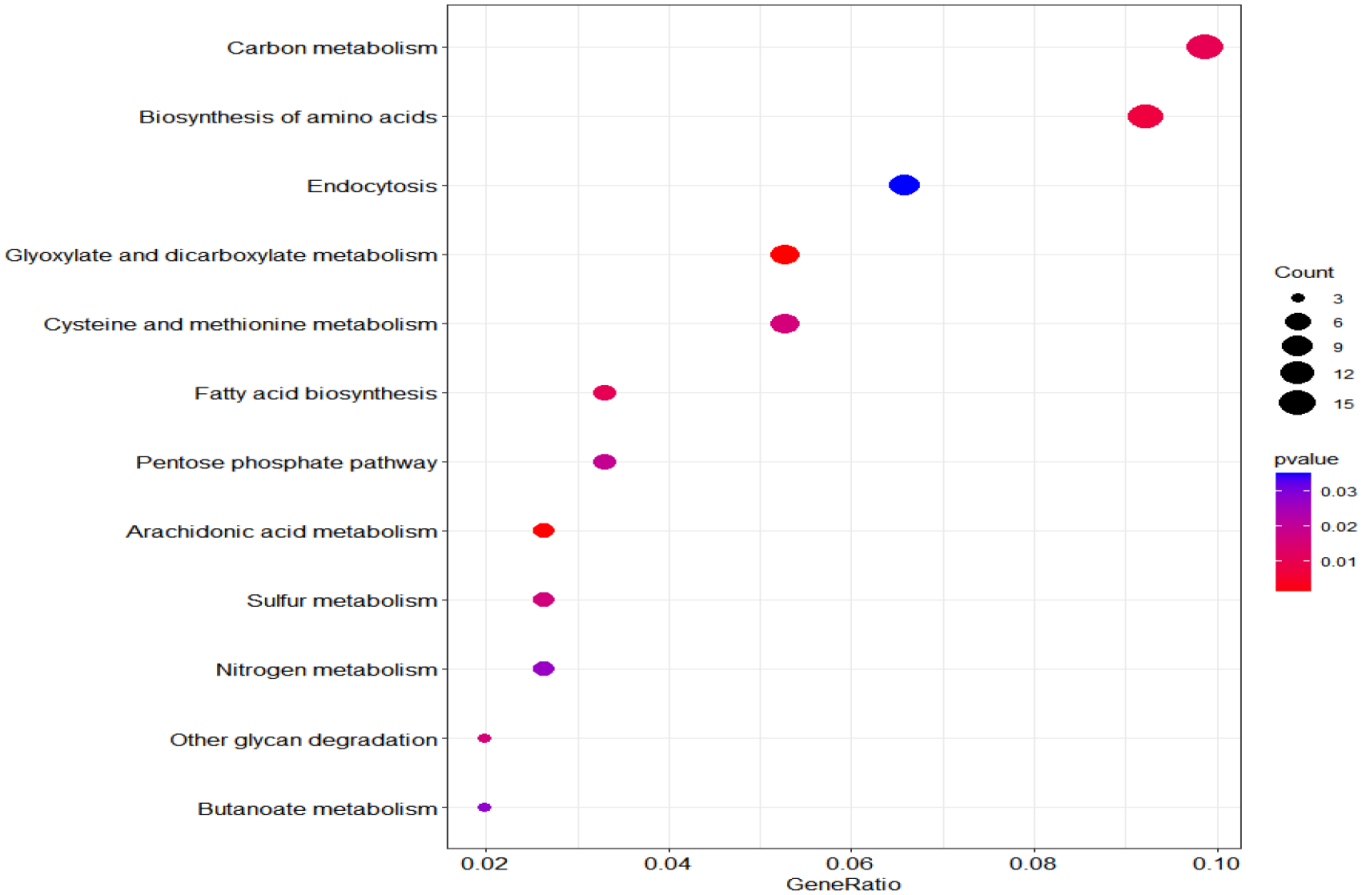

### DEPs screening

**Figure 5:**
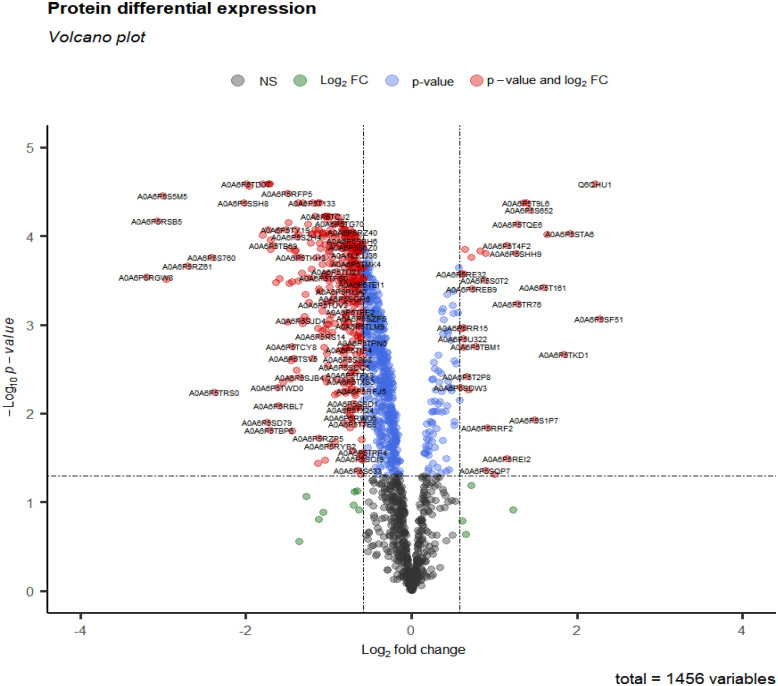
Volcano plot of differential expression protein in 28 days at 0°C.

After data preprocessing, 1456 proteins were obtained from group 3 (cluster 3, 4, 6, 9 and 11) at 28 days. Under the threshold of |log_2_FC| ≥ 0.58, adj.p-value < 0.05, total 411 DEPs were selected for subsequent analysis. Compared with CK28 group, 32 up-regulated and 379 down-regulated proteins (**Figure 5** and **Table S3**) (Liu et al., 2016).

### Identified protein KEGG functional annotation and enrichment analysis

The functions of proteins of group 3 were annotated based on the KEGG database. 411 uniprot IDs were converted to 409 ensemble gene IDs. 196 genes had KEGG annotations, a proportion of 47.68% of all proteins.

**Figure 6:**
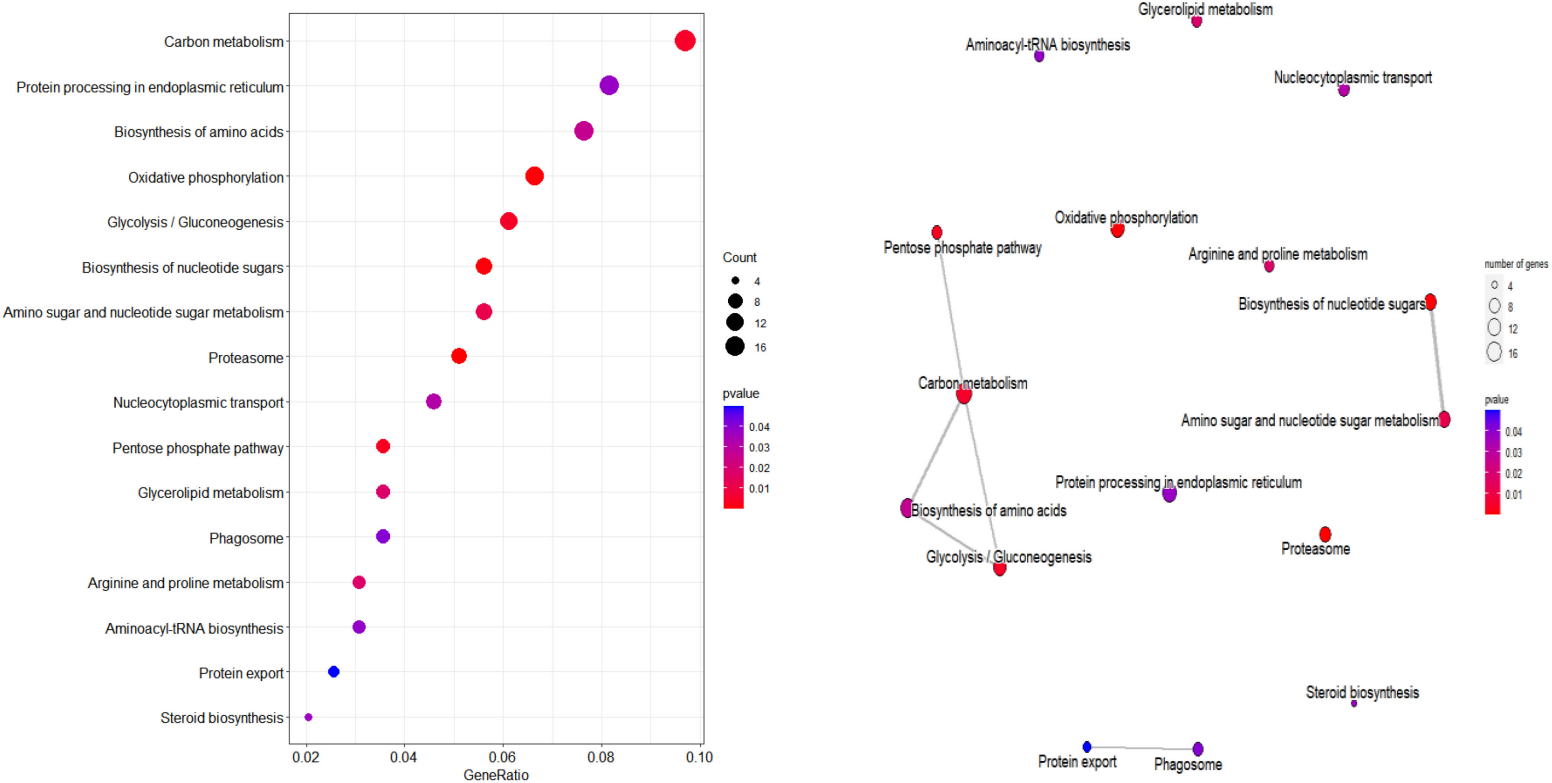
KEGG pathway plot and Emapplot of DEPs in group 3. According to the KEGG database: “GeneRatio” means the ratio of the number of differentially expressed genes under this pathway term to the number of annotated genes under the pathway term. The larger the value is, the greater the enrichment degree is in kegg and emapplot.

According to KEGG database, 196 proteins were enriched into 16 biological pathways (p-value<0.05) (**Figure 6**). The pathways belonged to energy metabolism, genetic information processing, environmental information processing, and cellular processes. The most abundant proteins were involved in energy metabolism (4 pathways) which were belonging to the metabolism, followed by the translation (2 pathways) and folding, sorting and degradation (2 pathways) which belonged to genetic information processing, and thirdly, signal transduction (1 pathway) which belonging to environmental information processing, in addition, transport and catabolism (1 pathway) which belonging to cellular processes (**Table 3**).

**Table 3:** KEGG pathway of group 3 (**Figure 6**).

KEGG name/ID	Gene name	Uniprot	Protein name/description	logFC
Pentose phosphate pathway (pavi00030)	*LOC110759612*	A0A6P5ST42	6-phosphogluconate dehydrogenase, decarboxylating 3	-0.69
	*LOC110749634*	A0A6P5RR27	probable 6-phosphogluconolactonase 4, chloroplastic	-0.70
	*LOC110768684*	A0A6P5TMK4	uncharacterized protein LOC110768684	-0.67
	*LOC110753532*	A0A6P5S8M0	fructose-bisphosphate aldolase 1, cytoplasmic	-0.69
	*LOC110758049*	A0A6P5SHP0	pyrophosphate–fructose 6-phosphate 1-phosphotransferase subunit alpha	-0.69
	*LOC110750379*	A0A6P5RYZ7	probable 6-phosphogluconolactonase 1	-0.69
	*LOC110767662*	A0A6P5THS0	phosphoglucomutase, chloroplastic	-0.63
Glycolysis/Gluconeogenesis (pavi00010)	*LOC110759242*	A0A6P5STH3	phosphoglycerate kinase 3, cytosolic	-0.65
	*LOC110750545*	A0A6P5RXW6	aldehyde dehydrogenase family 3 member H1 isoform X2	-0.61
	*LOC110760963*	A0A6P5SZC9	LOW QUALITY PROTEIN: pyruvate kinase isozyme A, chloroplastic-like	-0.78
	*LOC110749720*	A0A6P5RWU3	multiple inositol polyphosphate phosphatase 1-like isoform X1	-0.71
	*LOC110753532*	A0A6P5S8M0	fructose-bisphosphate aldolase 1, cytoplasmic	-0.69
	*LOC110762488*	A0A6P5SVE9	cytosolic enolase 3	-0.70
	*LOC110766537*	A0A6P5TEI1	aldo-keto reductase family 4 member C10-like	-0.61
	*LOC110758049*	A0A6P5SHP0	pyrophosphate–fructose 6-phosphate 1-phosphotransferase subunit alpha	-0.69
	*LOC110759031*	A0A6P5SSU7	pyruvate kinase, cytosolic isozyme	-0.67
	*LOC110752150*	A0A6P5S4L3	glyceraldehyde-3-phosphate dehydrogenase, cytosolic-like	-0.66
	*LOC110767662*	A0A6P5THS0	phosphoglucomutase, chloroplastic	-0.63
	*LOC110770067*	A0A6P5TS82	aldehyde dehydrogenase family 3 member H1-like	-0.80
Oxidative phosphorylation (pavi00190)	*LOC110758582*	A0A6P5SGU2	V-type proton ATPase subunit H	-1.03
	*LOC110770218*	A0A6P5TSZ9	NADH dehydrogenase [ubiquinone] iron-sulfur protein 8, mitochondrial	-1.17
	*LOC110766344*	A0A6P5TDA1	V-type proton ATPase subunit F	-0.79
	*LOC110744901*	A0A6P5RFK6	ATP synthase subunit O, mitochondrial	-0.74
	*LOC110766094*	A0A6P5TD27	cytochrome c1-2, heme protein, mitochondrial	-0.79
	*LOC110750913*	A0A6P5RZ61	NADH dehydrogenase [ubiquinone] 1 alpha subcomplex subunit 13-A	-2.71
	*LOC110753052*	A0A6P5RXN7	cytochrome b-c1 complex subunit 9-like	-0.69
	*LOC110764394*	A0A6P5T7A6	V-type proton ATPase subunit C	-0.59
	*LOC110764920*	A0A6P5T963	NADH dehydrogenase [ubiquinone] 1 alpha subcomplex subunit 6	-0.77
	*LOC110757961*	A0A6P5SNV3	external alternative NAD(P)H-ubiquinone oxidoreductase B3, mitochondrial	-0.79
	*LOC110769121*	A0A6P5TM74	cytochrome b-c1 complex subunit 7-2	-1.46
	*LOC110768696*	A0A6P5TMD9	cytochrome b-c1 complex subunit Rieske-4, mitochondrial-like	-0.68
	*LOC110771972*	A0A6P5TXY8	NADH dehydrogenase [ubiquinone] 1 alpha subcomplex subunit 1	-0.71
Glycerolipid metabolism (pavi00561)	*LOC110759292*	A0A6P5SS33	digalactosyldiacylglycerol synthase 2, chloroplastic isoform X1	-0.92
	*LOC110750545*	A0A6P5RXW6	aldehyde dehydrogenase family 3 member H1 isoform X2	-0.61
	*LOC110753288*	A0A6P5RYK4	lysophospholipid acyltransferase 1-like	-1.26
	*LOC110766537*	A0A6P5TEI1	aldo-keto reductase family 4 member C10-like	-0.61
	*LOC110770067*	A0A6P5TS82	aldehyde dehydrogenase family 3 member H1-like	-0.80
	*LOC110771484*	A0A6P5TWD0	1-acyl-sn-glycerol-3-phosphate acyltransferase 2	-1.61
	*LOC110760091*	A0A6P5SUX0	D-glycerate 3-kinase, chloroplastic	-0.73
Amino sugar and nucleotide sugar metabolism (pavi00520)	*LOC110771554*	A0A6P5TXP5	glucose-1-phosphate adenylyltransferase large subunit, chloroplastic/amyloplastic-like	-1.96
	*LOC110762082*	A0A6P5T133	UDP-glucuronic acid decarboxylase 6 isoform X1	-1.20
	*LOC110756609*	A0A6P5SHI4	UDP-glucuronic acid decarboxylase 5-like	-1.07
	*LOC110763904*	A0A6P5T5R8	putative GDP-L-fucose synthase 2	-0.80
	*LOC110747696*	A0A6P5RR53	bifunctional dTDP-4-dehydrorhamnose 3,5-epimerase/dTDP-4-dehydrorhamnose reductase	-0.65
	*LOC110753272*	A0A6P5S5E6	bifunctional UDP-glucose 4-epimerase and UDP-xylose 4-epimerase 1	-0.66
	*LOC110769416*	A0A6P5TPT2	NADH–cytochrome b5 reductase 1	-0.87
	*LOC110757714*	A0A6P5SGI4	GDP-mannose 4,6 dehydratase 1	-0.72
	*LOC110753405*	A0A6P5S300	alpha-1,4-glucan-protein synthase [UDP-forming] 2	-0.64
	*LOC110767344*	A0A6P5THH5	UDP-N-acetylglucosamine diphosphorylase 1	-1.08
	*LOC110767662*	A0A6P5THS0	phosphoglucomutase, chloroplastic	-0.63
Biosynthesis of nucleotide sugars (pavi01250)	*LOC110771554*	A0A6P5TXP5	glucose-1-phosphate adenylyltransferase large subunit, chloroplastic/amyloplastic-like	-1.96
	*LOC110762082*	A0A6P5T133	UDP-glucuronic acid decarboxylase 6 isoform X1	-1.20
	*LOC110756609*	A0A6P5SHI4	UDP-glucuronic acid decarboxylase 5-like	-1.07
	*LOC110763904*	A0A6P5T5R8	putative GDP-L-fucose synthase 2	-0.80
	*LOC110747696*	A0A6P5RR53	bifunctional dTDP-4-dehydrorhamnose 3,5-epimerase/dTDP-4-dehydrorhamnose reductase	-0.65
	*LOC110753272*	A0A6P5S5E6	bifunctional UDP-glucose 4-epimerase and UDP-xylose 4-epimerase 1	-0.66
	*LOC110755885*	A0A6P5SAH1	2-dehydro-3-deoxyphosphooctonate aldolase 1-like	-0.59
	*LOC110757714*	A0A6P5SGI4	GDP-mannose 4,6 dehydratase 1	-0.72
	*LOC110753405*	A0A6P5S300	alpha-1,4-glucan-protein synthase [UDP-forming] 2	-0.64
	*LOC110767344*	A0A6P5THH5	UDP-N-acetylglucosamine diphosphorylase 1	-1.08
	*LOC110767662*	A0A6P5THS0	phosphoglucomutase, chloroplastic	-0.63
Arginine and proline metabolism (pavi00330)	*LOC110771241*	A0A6P5TVM3	arginase 1, mitochondrial	-0.76
	*LOC110750545*	A0A6P5RXW6	aldehyde dehydrogenase family 3 member H1 isoform X2	-0.61
	*LOC110757265*	A0A6P5SLA2	proline iminopeptidase isoform X1	-0.83
	*LOC110752072*	A0A6P5RYV2	delta-1-pyrroline-5-carboxylate dehydrogenase 12A1, mitochondrial	-0.69
	*LOC110765925*	A0A6P5TCE0	probable polyamine oxidase 2	-1.59
	*LOC110770067*	A0A6P5TS82	aldehyde dehydrogenase family 3 member H1-like	-0.80
Biosynthesis of amino acids (pavi01230)	*LOC110771241*	A0A6P5TVM3	arginase 1, mitochondrial	-0.76
	*LOC110759242*	A0A6P5STH3	phosphoglycerate kinase 3, cytosolic	-0.65
	*LOC110768015*	A0A6P5TJN5	acetylornithine deacetylase	-0.73
	*LOC110763488*	A0A6P5T3N7	tryptophan synthase alpha chain	-1.22
	*LOC110760963*	A0A6P5SZC9	LOW QUALITY PROTEIN: pyruvate kinase isozyme A, chloroplastic-like	-0.78
	*LOC110773697*	A0A6P5U464	serine acetyltransferase 1, chloroplastic-like	-0.65
	*LOC110751150*	A0A6P5S1I7	serine acetyltransferase 5	0.89
	*LOC110768684*	A0A6P5TMK4	uncharacterized protein LOC110768684	-0.67
	*LOC110753532*	A0A6P5S8M0	fructose-bisphosphate aldolase 1, cytoplasmic	-0.69
	*LOC110762488*	A0A6P5SVE9	cytosolic enolase 3	-0.70
	*LOC110758653*	A0A6P5SRD7	3-dehydroquinate synthase, chloroplastic	-0.61
	*LOC110759031*	A0A6P5SSU7	pyruvate kinase, cytosolic isozyme	-0.67
	*LOC110752150*	A0A6P5S4L3	glyceraldehyde-3-phosphate dehydrogenase, cytosolic-like	-0.66
	*LOC110768524*	A0A6P5TLM9	diaminopimelate decarboxylase 1, chloroplastic-like	-0.61
	*LOC110754219*	A0A6P5SBM9	threonine dehydratase biosynthetic, chloroplastic	-0.66
Nucleocytoplasmic transport (pavi03013)	*LOC110771294*	A0A6P5TU18	elongation factor 1-alpha	-1.73
	*LOC110749570*	A0A6P5RQU6	importin subunit beta-1	-0.74
	*LOC110754597*	A0A6P5SCU9	eukaryotic initiation factor 4A-3	-0.73
	*LOC110763739*	A0A6P5T551	importin subunit alpha-2	-0.96
	*LOC110766679*	A0A6P5TEI0	importin subunit beta-1-like	
	*LOC110765688*	A0A6P5TC31	importin-4	-1.00
	*LOC110767294*	A0A6P5THD6	importin subunit beta-1-like	-1.39
	*LOC110750711*	A0A6P5RW06	importin subunit alpha-9	-0.73
	*LOC110770330*	A0A6P5TTE8	LOW QUALITY PROTEIN: RAN GTPase-activating protein 2	-0.71
Steroid biosynthesis (pavi00100)	*LOC110768162*	A0A6P5TKH3	24-methylenesterol C-methyltransferase 2	-1.34
	*LOC110750884*	A0A6P5S0I9	cycloartenol synthase 2	-1.38
	*LOC110766031*	A0A6P5TCY8	probable 3-beta-hydroxysteroid-Delta(8),Delta(7)-isomerase	-1.44
	*LOC110768705*	A0A6P5TME5	sterol 14-demethylase	-0.77
Protein processing in endoplasmic reticulum (pavi04141)	*LOC110751012*	A0A6P5RQI0	dolichyl-diphosphooligosaccharide–protein glycosyltransferase subunit STT3A	-1.11897
	*LOC110762860*	A0A6P5SY54	probable protein disulfide-isomerase A6	-0.69
	*LOC110749749*	A0A6P5RV81	eukaryotic translation initiation factor 2 subunit alpha homolog	-0.73
	*LOC110764580*	A0A6P5T7Q6	SEC12-like protein 2	-0.74
	*LOC110749381*	A0A6P5RTW9	GTP-binding protein SAR1A-like	-0.79
	*LOC110754946*	A0A6P5S452	protein transport protein Sec61 subunit gamma-like	-1.06
	*LOC110745511*	A0A6P5RHB1	phospholipase A-2-activating protein	-0.80
	*LOC110745667*	A0A6P5RKT8	ERAD-associated E3 ubiquitin-protein ligase component HRD3A	-0.66
	*LOC110758163*	A0A6P5SPT7	ubiquitin thioesterase OTU1	-0.87
	*LOC110757706*	A0A6P5SMW9	heat shock protein 90-5, chloroplastic	-0.76
	*LOC110763918*	A0A6P5T5S9	GTP-binding protein SAR1A-like	-0.78
	*LOC110766523*	A0A6P5TEM1	hsp70 nucleotide exchange factor fes1-like	-0.59
	*LOC110744987*	A0A6P5R736	protein transport protein Sec61 subunit alpha-like	-0.65
	*LOC110767398*	A0A6P5THX5	dolichyl-diphosphooligosaccharide–protein glycosyltransferase subunit DAD1	-0.67
	*LOC110758594*	A0A6P5SPJ9	ERAD-associated E3 ubiquitin-protein ligase HRD1B-like	-1.02
	*LOC110750725*	A0A6P5RW23	dnaJ protein P58IPK homolog	0.69
Proteasome (pavi03050)	*LOC110752154*	A0A6P5RZ40	26S proteasome non-ATPase regulatory subunit 6 homolog	-0.71
	*LOC110751327*	A0A6P5RY55	26S proteasome non-ATPase regulatory subunit 11 homolog	-1.00
	*LOC110768516*	A0A6P5TKB5	26S proteasome regulatory subunit S10B homolog B isoform X1	-0.85
	*LOC110758254*	A0A6P5SIC0	proteasome subunit alpha type-7	-0.75
	*LOC110761611*	A0A6P5T0R9	26S proteasome non-ATPase regulatory subunit 2 homolog A	-0.68
	*LOC110759896*	A0A6P5SVN7	26S proteasome regulatory subunit 8 homolog A	-0.62
	*LOC110761950*	A0A6P5STQ4	26S proteasome non-ATPase regulatory subunit 14 homolog	-0.69
	*LOC110750779*	A0A6P5RPM8	26S proteasome regulatory subunit 6B homolog	-0.61
	*LOC110752287*	A0A6P5S1R5	26S proteasome non-ATPase regulatory subunit 13 homolog B	-0.72
	*LOC110764158*	A0A6P5T6N7	26S proteasome non-ATPase regulatory subunit 12 homolog A-like	-0.79
Aminoacyl-tRNA biosynthesis (pavi00970)	*LOC110760913*	A0A6P5SQ42	LOW QUALITY PROTEIN: tyrosine–tRNA ligase 1, cytoplasmic-like	-1.21
	*LOC110767207*	A0A6P5TH00	tryptophan–tRNA ligase, cytoplasmic	-0.63
	*LOC110759739*	A0A6P5SN62	serine–tRNA ligase	-0.81
	*LOC110772028*	A0A6P5TYJ2	asparagine–tRNA ligase, cytoplasmic 1-like isoform X1	-0.87
	*LOC110764100*	A0A6P5T667	cysteine–tRNA ligase 2, cytoplasmic	-0.82
	*LOC110765644*	A0A6P5TBP6	tyrosine–tRNA ligase, chloroplastic/mitochondrial	-1.71
Phagosome (pavi04145)	*LOC110758582*	A0A6P5SGU2	V-type proton ATPase subunit H	-1.03
	*LOC110766344*	A0A6P5TDA1	V-type proton ATPase subunit F	-0.79
	*LOC110754946*	A0A6P5S452	protein transport protein Sec61 subunit gamma-like	-1.06
	*LOC110764394*	A0A6P5T7A6	V-type proton ATPase subunit C	-0.59
	*LOC110764808*	A0A6P5T8I7	25.3 kDa vesicle transport protein	-1.03
	*LOC110773403*	A0A6P5U2R9	tubulin alpha-3 chain	-0.82
	*LOC110744987*	A0A6P5R736	protein transport protein Sec61 subunit alpha-like	-0.65
Protein export (pavi03060)	*LOC110748656*	A0A6P5RMW7	signal recognition particle receptor subunit beta-like	-0.79
	*LOC110754946*	A0A6P5S452	protein transport protein Sec61 subunit gamma-like	-1.06
	*LOC110770381*	A0A6P5TSV5	signal peptidase complex catalytic subunit SEC11A	-1.42
	*LOC110744987*	A0A6P5R736	protein transport protein Sec61 subunit alpha-like	-0.65
	*LOC110757068*	A0A6P5SKK6	signal recognition particle subunit SRP68	-1.13
Carbon metabolism (pavi01200)	*LOC110753798*	A0A6P5S0B5	glycerate dehydrogenase	-1.19
	*LOC110759612*	A0A6P5ST42	6-phosphogluconate dehydrogenase, decarboxylating 3	-0.69
	*LOC110759242*	A0A6P5STH3	phosphoglycerate kinase 3, cytosolic	-0.65
	*LOC110760963*	A0A6P5SZC9	LOW QUALITY PROTEIN: pyruvate kinase isozyme A, chloroplastic-like	-0.78
	*LOC110749634*	A0A6P5RR27	probable 6-phosphogluconolactonase 4, chloroplastic	-0.70
	*LOC110773146*	A0A6P5U2L1	S-formylglutathione hydrolase	-0.61
	*LOC110773697*	A0A6P5U464	serine acetyltransferase 1, chloroplastic-like	-0.65
	*LOC110751150*	A0A6P5S1I7	serine acetyltransferase 5	0.89
	*LOC110768684*	A0A6P5TMK4	uncharacterized protein LOC110768684	-0.67
	*LOC110753532*	A0A6P5S8M0	fructose-bisphosphate aldolase 1, cytoplasmic	-0.69
	*LOC110762488*	A0A6P5SVE9	cytosolic enolase 3	-0.70
	*LOC110752818*	A0A6P5S118	dihydrolipoyllysine-residue succinyltransferase component of 2-oxoglutarate dehydrogenase complex 2, mitochondrial-like	-0.64
	*LOC110745262*	A0A6P5RGN9	malate dehydrogenase, glyoxysomal-like	-1.11
	*LOC110759031*	A0A6P5SSU7	pyruvate kinase, cytosolic isozyme	-0.67
	*LOC110752150*	A0A6P5S4L3	glyceraldehyde-3-phosphate dehydrogenase, cytosolic-like	-0.66
	*LOC110753434*	A0A6P5S955	ribulose bisphosphate carboxylase small chain clone 512-like	-0.66
	*LOC110750379*	A0A6P5RYZ7	probable 6-phosphogluconolactonase 1	-0.69
	*LOC110754219*	A0A6P5SBM9	threonine dehydratase biosynthetic, chloroplastic	-0.66
	*LOC110760091*	A0A6P5SUX0	D-glycerate 3-kinase, chloroplastic	-0.73

### DEPs verification

**Figure 7:**
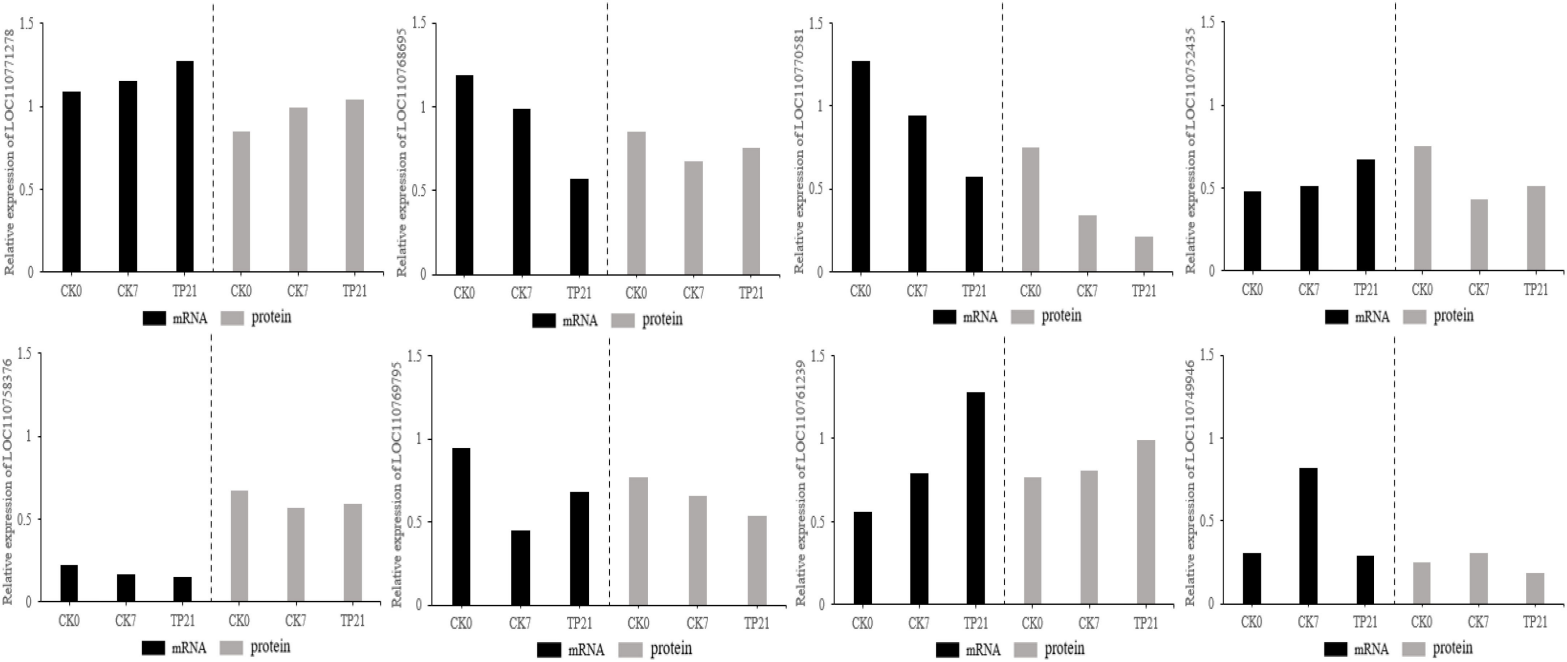
Verification of genes of protein using qRT-PCR.

To determine whether the levels of transcription and translation were correlated, the proteome data were checked at the transcription level. Results from qRT-PCR revealed that the expressions at the transcription level for most of the genes were similar to the results of cherry protein abundance among the eight randomly selected proteins (**Figure 7**).

## Discussion

Cherry fruit undergoes a rapid decay and disintegration process that involves several differential expression proteins during storage. In the study, CK disintegrated rapidly at 28 days of storage, while TP was more stable at TP vs CK, and the differential proteins were mainly related to energy metabolism, protein synthesis and modification, amino acid and cell wall metabolism.

### Proteins associated with energy metabolism

The DEPs related to energy in TP vs CK group were involved in pentose phosphate pathway, glycolysis/gluconeogenesis, oxidative phosphorylation. Glycolysis refers to the process in which glucose is decomposed to produce pyruvate and provide energy under anaerobic conditions (Serina et al., 2019). Gluconeogenesis refers to the process in which non sugar substances such as pyruvate, pyruvic acid etc are used as precursors to synthesize glucose. There is a close relationship between gluconeogenesis and glycolysis. If glycolysis is active, gluconeogenesis is limited, conversely, if the key enzyme of glycolysis is inhibited, the acting enzyme of gluconeogenesis will be promoted (Quiroga et al., 2022). Glycolysis plays an important role in carbohydrate metabolism, and in this study, glyceraldehyde-3-phosphate dehydrogenase, phosphoglycerate kinase, pyruvate kinase, and fructose-bisphosphate aldolase screened were a key enzyme in the process of glycolysis and were up-regulated in CK group. The up-regulated expression of hexokinase gene can promote the weakening of gluconeogenesis pathway, leading to the enhancement of pentose phosphate pathway and glycolysis pathway. NADH dehydrogenase, ATP synthase, cytochrome, V-type proteon ATPase in oxidative phosphorylation were up-regualted expression, this indicate that the more energy was produced in CK group, and the subsequent metabolic process should be at a high level.

### Proteins associated with translation and folding, sorting and degradation

Proteins, an important part of the enzymes in many plants, providing nutrition and energy for plant storage, participating in the regulation of various plant metabolic processes, are closely related to plant metabolism, and stress resistance. In this study, This pathways (arginine and proline metabolism, aminoacyl-tRNA biosynthesis, protein processing in endoplasmic reticulum, protein export, nucleocytoplasmic transport, proteasome) related to DEPs belong to animo acid biosynthesis, translation, and folding, sorting and degradation. The final product of biological function is protein, and amino acids are a class of crucial natural molecules for building a variety of diverse peptides and proteins, which can also take part in the formation of metabolites in organisms (Huang et al., 2020; Huang et al., 2021). When plants are in a stress environment, in order to better adapt to this environment, amino acids in cells will respond to a certain extent. In the study, the results showed that the synthesis of amino acids of cherry was almost down-regulated after ozone treatment, indicating that the lower synthesis of amino acids may be a common response under the action of ozone, while the synthesis and expression of most amino acids in CK group were up-regulated, which further indicated that the CK group was more sensitive in responding to the process of ozone. Sufficient amino acids were provided for metabolic pathways, aminoacyl-tRNA biosynthesis and protein processing in endoplasmic reticulum are important metabolic pathways for protein formation. Aminoacylation of tRNAs was the critical step of protein biosynthesis, amino acids were first activated and then transfered to the tRNA (Cramer et al., 1991). In protein biosynthesis, aminoacyl-tRNA plays a crucial role in the process of transferring amino acids to the carboxyl end of peptide chain. Meanwhile, in our work, proteasome, protein processing in endoplasmic reticulum was associated with ozone-treated cherry. It shows that ozone treatment affected the expression of some proteins.

### Proteins associated with cell wall metabolism

In our stuty, these DEPs were also enriched in several pathways such as biosynthesis of nucleotide sugars, amino sugar and nucleotide sugar metabolism (**Figure S3**). The most common forms of amino sugars are glucosamine and galactosamine, and amino sugar metabolism is closely related to plant growth and development and environmental adaptability. Nucleotide sugar is an activated form of carbohydrate synthesis or mutual conversion. Ozone treatment may affect the expression of various metabolic enzymes of amino sugar and nucleotide sugar in cherry, and play a role in regulating the quality of cherry, especially firmness. Some enzymes in the amino sugar and nucleotide sugar metabolism pathway also participate in cell wall metabolism, such as chitinase, endogenous chitinase, mannose-1-phosphate guanyltransferase (MPG) and UDP glucuronic acid decarboxylase (UXS). Therefore, with the fruit at the ozone treatment, the cherry fruit degradation slowed down and attained a stable metabolism situation.

### Proteins associated with lipid metabolism

These DEPs were also enriched in several pathways such as glycerolipid metabolism, steroid biosynthesis (**Figure S3**). A specific fruit can be recognized by the combination of volatile organic chemicals that give sweet cherries their distinctive flavor. Volatiles are derived from metabolites like fatty acids and other compounds. Glycerolipids are the most abundant lipids in higher plants (Qin et al., 2020). Plant lipids include lipids, membrane lipids, signal molecules, photosynthetic pigments, essential oils, plant hormones and plant surface protective substances. They play an important role in plant growth, storage and stress response, and are widely involved in different biological processes. In addition to being an important structural material of organisms, lipids include the lipid bilayer of cell membrane and protective lipids on the surface of plant tissues and organs. Lipids are also very important physiological active substances and signal molecules in plants. For example, phosphatidic acid, brassinolide and phosphatidylinositol derivatives are widely involved in various biological processes in plants (Zheng and Li, 2017; Qin et al., 2020). Lipid is also an efficient energy storage material in plants. The energy stored by lipid per unit weight is much higher than that of carbohydrate and protein. Lipid storage, such as triglyceride, can provide sufficient energy and carbon source for biological metabolism. Membrane is sensitive to environmental changes, and abiotic stress directly affects membrane performance. In addition, glycerides are the main components of the membrane. Adjusting the composition, unsaturation and acyl chain length of the membrane also enables plants to maintain the integrity and fluidity of the membrane under environmental stress. Sequential action by acyl-releasing lipases and acyltransferases can replace the acyl groups of the bulk membrane glycerolipids while modifying the biophysical characterisitics of each lipid molecules to maintain the membrane bilayer structure. Free fatty acids (FAs) and a less lipophilic molecule, such as a lysophospholipid, are produced when acyl-releasing glycerolipid lipases hydrolyze the acyl groups from a glycerolipid molecule (Wang et al., 2019; Kim, 2020). Meanwhile, fatty acids can improve the tolerance of plants and reduce the damage of abiotic stress to plants.

## Conclusion

In this study, 4557 master proteins were identified, and 3149 proteins were common to all groups. Mfuzz analyses revealed 3149 candidate proteins. KEGG annotation and enrichment analysis showed proteins related to carbohydrate and energy metabolism, protein, amino acids, and nucleotide sugar biosynthesis and degradation. The expressions on the transcription level of eight selected proteins by qRT-PCR validation were similar to the results of the protein level. Further study of the molecular structures of these proteins and their associated biological roles in cherry during storage.

## Data availability statement

The mass spectrometry proteomics data have been deposited to the ProteomeXchange Consortium (http://proteomecentral.proteomexchange.org) with the dataset identifier PXD037578.

## Author contributions

YZ was responsible for the design of the experiments, and interpretation of data. ZH and YZ performed the experiments. YZ wrote the manuscript. NZ and HJ collected relevant materials. CD determined compounds and improved the results. XC assist in raw data uploading. JY improve the discussion. CC contributed to funding acquisition. HG contributed to reviewing and editing the manuscript.. All authors contributed to the article and approved the submitted version.
