# Supplementary material for: Application of proteomics to determine the mechanism of ozone on sweet cherries (Prunus avium L.) by time-series analysis
Source: Front Plant Sci. 2023 Feb 9;14:1065465. doi: 10.3389/fpls.2023.1065465 (PMC9948404; doi:10.3389/fpls.2023.1065465)
Supplement: Supplementary file 1 [file DataSheet_1.docx]

Supplementary Material

Table S1 Physical and physiological traits of sweet cherry during storage and response to ozone

| **Parameters** | **Treatments** | **Storage time (days)** | | | | | | |
| --- | --- | --- | --- | --- | --- | --- | --- | --- |
|  |  | 0 | 7 | 14 | 21 | 28 | 35 | 42 |
| firmness | control | 8.78±0.59 | 7.88±0.02 | 6.91±0.29 | 6.23±0.27 | 5.53±0.64 | 5.33±0.95 | 5.14±0.96 |
|  | ozone | 8.78±0.59 | 9.32±0.06 | 8.74±0.31 | 8.07±0.29 | 7.52±0.35 | 7.25±0.46 | 6.89±0.79 |
| weight loss | control | 0 | 2.31±0.11 | 5.22±0.29 | 7.68±1.41 | 8.28±0.46 | 8.33±0.66 | 9.01±0.78 |
|  | ozone | 0 | 0 | 0.09±0.02 | 0.14±0.01 | 0.19±0.10 | 0.23±0.07 | 0.32±0.02 |
| rotting rate | control | 0 | 0 | 0 | 0 | 1.99±0.24 | 3.17±0.35 | 3.62±0.29 |
|  | ozone | 0 | 0 | 0 | 0 | 0 | 0 | 0 |
| TSS | control | 14.27±0.81 | 14.83±1.34 | 15.43±0.95 | 16.04±0.21 | 18.57±0.66 | 19.01±0.78 | 20.29±0.56 |
|  | ozone | 14.27±0.81 | 14.75±0.93 | 14.53±0.82 | 14.11±0.14 | 13.96±0.85 | 14.19±0.32 | 12.74±0.54 |
| TA | control | 0.82±0.23 | 0.63±0.10 | 0.57±0.06 | 0.53±0.04 | 0.53±0.14 | 0.49±0.65 | 0.45±0.23 |
|  | ozone | 0.82±0.23 | 0.78±0.09 | 0.71±0.05 | 0.71±0.06 | 0.68±0.62 | 0.69±0.95 | 0.60±0.29 |
| SOD | control | 1049.35±102.37 | 882.29±102.31 | 720.36±60.33 | 798.38±82.36 | 682.46±77.89 | 660.45±58.86 | 638.78±62.69 |
|  | ozone | 1049.35±102.37 | 855.86±74.21 | 1083.29±111.29 | 945.64±96.24 | 1119.22±124.32 | 1190.61±100.68 | 1260.36±138.36 |
| POD | control | 78.89±6.37 | 60.23±5.34 | 31.11±5.14 | 41.11±2.95 | 25.44±1.45 | 23.79±3.76 | 21.98±3.69 |
|  | ozone | 78.89±6.37 | 67.78±4.31 | 43.67±5.16 | 76.67±9.76 | 61.11±8.98 | 64.44±0.36 | 61.28±7.68 |
| APX | control | 0.29±0.15 | 0.27±0.03 | 0.23±0.13 | 0.27±0.05 | 0.24±0.04 | 0.21±0.08 | 0.19±0.06 |
|  | ozone | 0.29±0.15 | 0.28±0.08 | 0.31±0.01 | 0.31±0.07 | 0.31±0.07 | 0.31±0.05 | 0.28±0.05 |
| Vc | control | 11.52±1.25 | 9.81±0.77 | 8.69±1.12 | 6.31±0.85 | 5.91±0.98 | 5.11±0.61 | 4.59±0.66 |
|  | ozone | 11.52±1.25 | 11.33±0.60 | 10.93±0.98 | 10.14±0.90 | 9.43±0.72 | 8.51±0.77 | 7.98±0.89 |
| total flavonoid | control | 225.67±13.81 | 262.72±23.51 | 233.82±20.20 | 258.47±43.66 | 267.58±43.52 | 271.99±43.07 | 279.85±26.49 |
|  | ozone | 225.67±13.81 | 307.44±63.77 | 320.25±33.59 | 330.07±39.81 | 291.63±13.96 | 273.62±33.44 | 269.45±20.37 |
| total phenol | control | 425.28±93.29 | 450.94±23.51 | 457.38±53.68 | 474.98±60.22 | 497.93±55.19 | 513.66±46.39 | 520.42±33.88 |
|  | ozone | 425.28±93.29 | 497.71±44.49 | 517.53±21.87 | 554.89±45.29 | 589.22±38.21 | 610.38±12.69 | 622.39±28.91 |
| anthocyanins | control | 53.97±3.29 | 47.33±6.13 | 40.36±3.83 | 44.92±4.22 | 40.48±2.28 | 39.22±1.28 | 40.48±6.33 |
|  | ozone | 53.97±3.29 | 64.54±4.66 | 56.15±6.01 | 53.56±3.20 | 57.94±4.01 | 58.35±1.76 | 61.35±5.58 |

Data were expressed as mean ± standard deviation (n = 3).

Table S2 Result of proteins (Figure S1)

| sample | Number of Master proteins | Number of Common proteins |
| --- | --- | --- |
| CK00 | 3756 | 3149 |
| CK07 | 3779 |  |
| CK14 | 3789 |  |
| CK21 | 3760 |  |
| CK28 | 3800 |  |
| CK35 | 3643 |  |
| CK42 | 3601 |  |
| TP07 | 3809 |  |
| TP14 | 3751 |  |
| TP21 | 3753 |  |
| TP28 | 3744 |  |
| TP35 | 3725 |  |
| TP42 | 3731 |  |
| total | 4557 |  |

Table S3 Different expression proteins of group 3 (Figure 5)

| accession | logFC | P value | accession | logFC | P value |
| --- | --- | --- | --- | --- | --- |
| Q6QHU1 | 2.216705606 | 9.02E-08 | A0A6P5TD07 | -1.994790222 | 2.37E-08 |
| A0A6P5T9L6 | 1.392109193 | 3.52E-07 | A0A6P5SU06 | -1.800028882 | 4.41E-08 |
| A0A6P5U131 | 1.35880251 | 4.29E-07 | A0A6P5RQI1 | -1.71784341 | 7.44E-08 |
| A0A6P5S652 | 1.424316754 | 6.22E-07 | A0A6P5S9I9 | -1.727008236 | 9.34E-08 |
| A0A6P5TQE6 | 1.275769251 | 1.73E-06 | A0A6P5TG78 | -1.724783558 | 1.06E-07 |
| A0A6P5STA6 | 1.918048988 | 3.49E-06 | A0A6P5TXP5 | -1.962789359 | 1.32E-07 |
| A0A6P5RZ03 | 1.637392013 | 4.24E-06 | A0A6P5RFP5 | -1.495261111 | 1.82E-07 |
| A0A6P5T4F2 | 1.147929206 | 8.32E-06 | A0A6P5S5M5 | -3.004181435 | 2.14E-07 |
| A0A6P5S347 | 0.646456772 | 1.12E-05 | A0A6P5T133 | -1.20091492 | 3.20E-07 |
| A0A6P5SIU5 | 0.825369479 | 1.34E-05 | A0A6P5U1J2 | -1.364103245 | 4.62E-07 |
| A0A6P5SHH9 | 1.261972971 | 1.49E-05 | A0A6P5SSH8 | -2.022479007 | 4.68E-07 |
| A0A6P5S1I7 | 0.889538741 | 1.50E-05 | A0A6P5TK84 | -1.111166351 | 4.70E-07 |
| A0A6P5S5P9 | 0.718398994 | 1.77E-05 | A0A6P5TN15 | -1.128730134 | 4.86E-07 |
| A0A6P5RE32 | 0.607347227 | 3.50E-05 | A0A6P5U5A2 | -1.314150337 | 4.90E-07 |
| A0A6P5T1J0 | 0.62052236 | 3.65E-05 | A0A6P5TCJ2 | -1.035250384 | 8.34E-07 |
| A0A6P5S0T2 | 0.88200094 | 4.98E-05 | A0A6P5T8U1 | -0.912127347 | 8.80E-07 |
| A0A6P5T161 | 1.591923984 | 6.76E-05 | A0A6P5T492 | -1.169061125 | 9.50E-07 |
| A0A6P5REB9 | 0.730430114 | 7.26E-05 | A0A6P5SGU2 | -1.028275756 | 9.53E-07 |
| A0A6P5TR76 | 1.280442854 | 0.000127658 | A0A6P5TU86 | -1.04530007 | 9.72E-07 |
| A0A6P5SF51 | 2.276192941 | 0.000213714 | A0A6P5TKA5 | -0.992403002 | 9.91E-07 |
| A0A6P5RR15 | 0.628605777 | 0.000300531 | A0A6P5RQ62 | -0.880416439 | 1.05E-06 |
| A0A6P5TLX9 | 0.589923998 | 0.000316826 | A0A6P5RPK7 | -1.008620461 | 1.18E-06 |
| A0A6P5U322 | 0.624136827 | 0.000434804 | A0A6P5RSB5 | -3.073514875 | 1.26E-06 |
| A0A6P5TBM1 | 0.769500881 | 0.000552667 | A0A6P5S7F4 | -1.48962085 | 1.35E-06 |
| A0A6P5SCM5 | 0.624575699 | 0.00059042 | A0A6P5TG70 | -0.86089989 | 1.44E-06 |
| A0A6P5TKD1 | 1.845397165 | 0.000756886 | A0A0C5DBY5 | -0.854452238 | 1.48E-06 |
| A0A6P5T2P8 | 0.671403746 | 0.001562101 | A0A6P5TUU2 | -0.834449752 | 1.58E-06 |
| A0A6P5SDW3 | 0.595033323 | 0.002249477 | A0A6P5RT09 | -1.246625581 | 1.62E-06 |
| A0A6P5RW23 | 0.69014607 | 0.002489156 | A0A6P5RLN8 | -0.93059733 | 1.66E-06 |
| A0A6P5S1P7 | 1.488606024 | 0.006188458 | A0A6P5TGV0 | -0.926592747 | 1.77E-06 |
| A0A6P5RRF2 | 0.915746811 | 0.007945584 | A0A6P5TY19 | -1.524477634 | 2.08E-06 |
| A0A6P5REI2 | 1.153568959 | 0.019762988 | A0A6P5TJZ2 | -0.984092119 | 2.24E-06 |
| A0A6P5SQP7 | 0.893060007 | 0.028192503 | A0A6P5RXJ1 | -0.810301797 | 2.35E-06 |
| A0A6P5SZQ6 | 0.995734081 | 0.031706096 | A0A6P5RWN1 | -0.812673015 | 2.39E-06 |
|  |  |  | A0A6P5T9K1 | -0.838921823 | 2.43E-06 |
|  |  |  | A0A6P5TSZ9 | -1.169098164 | 2.50E-06 |
|  |  |  | A0A6P5SM41 | -0.816914405 | 2.50E-06 |
|  |  |  | A0A6P5SZ69 | -1.39778256 | 2.55E-06 |
|  |  |  | A0A6P5RQI0 | -1.118977173 | 2.58E-06 |
|  |  |  | A0A6P5TU18 | -1.734238992 | 2.60E-06 |
|  |  |  | A0A6P5SBY3 | -0.742493806 | 2.67E-06 |
|  |  |  | A0A6P5RYS6 | -1.602438694 | 2.77E-06 |
|  |  |  | A0A6P5RZ40 | -0.714217762 | 2.98E-06 |
|  |  |  | A0A6P5SHI4 | -1.068527421 | 3.07E-06 |
|  |  |  | A0A6P5TXD9 | -1.074190234 | 3.18E-06 |
|  |  |  | A0A6P5TUZ5 | -0.774321028 | 3.21E-06 |
|  |  |  | A0A6P5RMW7 | -0.798278873 | 3.35E-06 |
|  |  |  | A0A6P5RQU6 | -0.736281747 | 3.38E-06 |
|  |  |  | A0A6P5RS18 | -0.768904905 | 3.51E-06 |
|  |  |  | A0A6P5T3D7 | -1.139543889 | 3.61E-06 |
|  |  |  | A0A6P5TB28 | -0.982568525 | 3.63E-06 |
|  |  |  | A0A6P5S0B5 | -1.194755758 | 3.74E-06 |
|  |  |  | A0A6P5SQ42 | -1.205764406 | 3.92E-06 |
|  |  |  | A0A6P5SS33 | -0.920642276 | 3.95E-06 |
|  |  |  | A0A6P5RY55 | -1.005359593 | 4.04E-06 |
|  |  |  | A0A6P5TWR1 | -1.370456748 | 4.05E-06 |
|  |  |  | A0A6P5TZW8 | -1.063002992 | 4.19E-06 |
|  |  |  | A0A6P5R4T3 | -0.690231823 | 4.24E-06 |
|  |  |  | A0A6P5RZN7 | -1.795056643 | 4.41E-06 |
|  |  |  | A0A6P5TUZ0 | -0.674238086 | 4.58E-06 |
|  |  |  | A0A6P5S9D0 | -0.663296395 | 4.85E-06 |
|  |  |  | A0A6P5TDA1 | -0.798819461 | 4.85E-06 |
|  |  |  | A0A6P5S2H4 | -1.394559576 | 5.05E-06 |
|  |  |  | A0A6P5T5R8 | -0.804358758 | 5.12E-06 |
|  |  |  | A0A6P5TKB5 | -0.845985445 | 5.15E-06 |
|  |  |  | A0A6P5TVM3 | -0.760942421 | 5.27E-06 |
|  |  |  | A0A6P5SN68 | -0.751679978 | 5.28E-06 |
|  |  |  | A0A6P5TBE6 | -1.065611486 | 5.30E-06 |
|  |  |  | A0A6P5SIC0 | -0.748178139 | 5.32E-06 |
|  |  |  | A0A6P5T1M8 | -0.925161209 | 5.36E-06 |
|  |  |  | A0A6P5ST42 | -0.695175424 | 5.47E-06 |
|  |  |  | A0A6P5TEA6 | -1.700465168 | 5.94E-06 |
|  |  |  | A0A6P5SBH6 | -0.713063251 | 6.22E-06 |
|  |  |  | A0A6P5STH3 | -0.649319397 | 6.40E-06 |
|  |  |  | A0A6P5SKE5 | -0.978649234 | 6.47E-06 |
|  |  |  | A0A6P5TJN5 | -0.728539439 | 6.72E-06 |
|  |  |  | A0A6P5T3N7 | -1.217853344 | 6.89E-06 |
|  |  |  | A0A6P5TS03 | -0.598262518 | 7.11E-06 |
|  |  |  | A0A6P5SUH4 | -0.887317266 | 7.13E-06 |
|  |  |  | A0A6P5SM06 | -1.111558393 | 7.16E-06 |
|  |  |  | A0A6P5RPY9 | -0.645254537 | 7.33E-06 |
|  |  |  | A0A6P5TRB4 | -0.784303428 | 7.39E-06 |
|  |  |  | A0A6P5RFK6 | -0.740925292 | 7.68E-06 |
|  |  |  | A0A6P5TFG3 | -0.726300563 | 7.86E-06 |
|  |  |  | A0A6P5TFI6 | -0.863062977 | 7.86E-06 |
|  |  |  | A0A6P5TD27 | -0.791228723 | 8.05E-06 |
|  |  |  | A0A6P5TA88 | -0.79312758 | 8.17E-06 |
|  |  |  | A0A6P5RRX1 | -1.046779157 | 8.20E-06 |
|  |  |  | A0A6P5TB69 | -1.669226235 | 8.48E-06 |
|  |  |  | A0A6P5RWR2 | -0.963866937 | 8.60E-06 |
|  |  |  | T1P3T4 | -0.730101603 | 8.61E-06 |
|  |  |  | A0A6P5S6Z0 | -0.699373561 | 9.36E-06 |
|  |  |  | A0A6P5RXW6 | -0.613671944 | 9.55E-06 |
|  |  |  | A0A6P5SZC9 | -0.784697045 | 9.59E-06 |
|  |  |  | A0A6P5RQ55 | -0.996068774 | 9.69E-06 |
|  |  |  | A0A6P5SY54 | -0.69100051 | 9.70E-06 |
|  |  |  | A0A6P5SJ76 | -0.632040814 | 9.70E-06 |
|  |  |  | A0A6P5T0R9 | -0.682261986 | 9.77E-06 |
|  |  |  | A0A6P5RR27 | -0.700852515 | 9.86E-06 |
|  |  |  | A0A6P5S119 | -0.9154479 | 9.91E-06 |
|  |  |  | A0A6P5RV81 | -0.726029838 | 1.04E-05 |
|  |  |  | A0A6P5SLA2 | -0.831756658 | 1.06E-05 |
|  |  |  | A0A6P5RUV4 | -1.103459437 | 1.06E-05 |
|  |  |  | A0A6P5TTT1 | -1.480659549 | 1.06E-05 |
|  |  |  | A0A6P5RL47 | -0.645670123 | 1.08E-05 |
|  |  |  | A0A6P5RY44 | -0.656151898 | 1.10E-05 |
|  |  |  | A0A6P5T7P9 | -0.840835427 | 1.10E-05 |
|  |  |  | A0A6P5T1M6 | -0.587434643 | 1.11E-05 |
|  |  |  | A0A6P5TRX3 | -0.681579779 | 1.13E-05 |
|  |  |  | A0A6P5SKY3 | -0.591759502 | 1.15E-05 |
|  |  |  | A0A6P5U251 | -1.69810396 | 1.16E-05 |
|  |  |  | A0A6P5T6D1 | -0.694176409 | 1.18E-05 |
|  |  |  | A0A6P5RR53 | -0.649873522 | 1.20E-05 |
|  |  |  | A0A2S1CES5 | -1.402427828 | 1.21E-05 |
|  |  |  | A0A6P5T888 | -0.689231279 | 1.23E-05 |
|  |  |  | A0A6P5U2L1 | -0.607668979 | 1.26E-05 |
|  |  |  | A0A6P5RVC6 | -0.989066364 | 1.27E-05 |
|  |  |  | A0A6P5R957 | -0.729007331 | 1.29E-05 |
|  |  |  | A0A6P5SNR0 | -0.612546748 | 1.30E-05 |
|  |  |  | A0A6P5SBW6 | -1.409311538 | 1.32E-05 |
|  |  |  | A0A6P5SDE7 | -1.002321179 | 1.34E-05 |
|  |  |  | A0A6P5T2Q0 | -0.634121357 | 1.35E-05 |
|  |  |  | A0A6P5RVS5 | -0.77736727 | 1.36E-05 |
|  |  |  | A0A6P5S5E6 | -0.655336706 | 1.38E-05 |
|  |  |  | A0A6P5U464 | -0.651569709 | 1.43E-05 |
|  |  |  | A0A1L5JJ36 | -0.690534998 | 1.57E-05 |
|  |  |  | A0A6P5SCU9 | -0.729307683 | 1.58E-05 |
|  |  |  | A0A6P5T7Q6 | -0.744184899 | 1.68E-05 |
|  |  |  | A0A6P5RIR2 | -1.046983139 | 1.70E-05 |
|  |  |  | A0A6P5S760 | -2.417474 | 1.71E-05 |
|  |  |  | A0A6P5TKH3 | -1.338372061 | 1.72E-05 |
|  |  |  | F2VR43 | -1.144395249 | 1.74E-05 |
|  |  |  | A0A6P5TAT0 | -0.699331276 | 1.75E-05 |
|  |  |  | A0A6P5S0I9 | -1.377076327 | 1.79E-05 |
|  |  |  | A0A6P5T6S4 | -0.7635384 | 1.79E-05 |
|  |  |  | A0A6P5SR52 | -0.838556133 | 1.81E-05 |
|  |  |  | A0A6P5T551 | -0.964415712 | 1.82E-05 |
|  |  |  | A0A6P5RH40 | -0.702775305 | 1.97E-05 |
|  |  |  | A0A6P5SHC7 | -0.704410485 | 2.04E-05 |
|  |  |  | A0A6P5T3Y3 | -0.661814876 | 2.06E-05 |
|  |  |  | A0A6P5T8W8 | -0.782074001 | 2.18E-05 |
|  |  |  | A0A6P5TMK4 | -0.670160175 | 2.26E-05 |
|  |  |  | A0A6P5TPT2 | -0.873247519 | 2.27E-05 |
|  |  |  | A0A6P5TI49 | -0.99029466 | 2.28E-05 |
|  |  |  | A0A6P5RJQ4 | -0.718584137 | 2.28E-05 |
|  |  |  | A0A6P5RTX7 | -0.669702836 | 2.29E-05 |
|  |  |  | A0A6P5S850 | -0.945762616 | 2.29E-05 |
|  |  |  | A0A6P5TSQ3 | -1.078805387 | 2.37E-05 |
|  |  |  | A0A6P5TH00 | -0.629830683 | 2.51E-05 |
|  |  |  | A0A6P5RMT5 | -0.75958997 | 2.53E-05 |
|  |  |  | A0A6P5RZ61 | -2.70703016 | 2.54E-05 |
|  |  |  | A0A6P5TCS7 | -0.722230451 | 2.55E-05 |
|  |  |  | A0A6P5SN62 | -0.805040478 | 2.71E-05 |
|  |  |  | A0A6P5TBU5 | -0.617755481 | 2.74E-05 |
|  |  |  | A0A6P5S263 | -0.637305571 | 2.79E-05 |
|  |  |  | A0A6P5RWU3 | -0.708446621 | 2.87E-05 |
|  |  |  | A0A6P5RL45 | -0.588609196 | 2.87E-05 |
|  |  |  | A0A6P5RXN7 | -0.693737252 | 2.89E-05 |
|  |  |  | A0A6P5SYY0 | -0.587756664 | 2.93E-05 |
|  |  |  | A0A6P5S2Z9 | -1.21262406 | 2.96E-05 |
|  |  |  | A0A6P5RI01 | -0.614943069 | 3.02E-05 |
|  |  |  | A0A6P5TD21 | -0.903973851 | 3.16E-05 |
|  |  |  | A0A6P5SYH6 | -0.740604118 | 3.25E-05 |
|  |  |  | A0A6P5S8M0 | -0.698692814 | 3.28E-05 |
|  |  |  | A0A6P5SVN7 | -0.616708084 | 3.37E-05 |
|  |  |  | A0A6P5RWA2 | -1.323920503 | 3.38E-05 |
|  |  |  | A0A6P5RJQ8 | -1.046721838 | 3.53E-05 |
|  |  |  | A0A6P5U1F5 | -0.998628472 | 3.58E-05 |
|  |  |  | A0A6P5RTW9 | -0.799912292 | 3.59E-05 |
|  |  |  | A0A6P5S452 | -1.059693205 | 3.97E-05 |
|  |  |  | A0A6P5SVE9 | -0.70206457 | 4.00E-05 |
|  |  |  | A0A6P5SV44 | -0.628861111 | 4.00E-05 |
|  |  |  | A0A6P5RYV2 | -0.688472656 | 4.01E-05 |
|  |  |  | A0A6P5SRZ0 | -0.880399074 | 4.03E-05 |
|  |  |  | A0A6P5RGW8 | -3.196634894 | 4.09E-05 |
|  |  |  | A0A6P5TWR7 | -0.642375662 | 4.20E-05 |
|  |  |  | A0A6P5T8Y8 | -0.821279191 | 4.21E-05 |
|  |  |  | A0A6P5TFS0 | -1.054887388 | 4.30E-05 |
|  |  |  | A0A6P5S286 | -0.708158031 | 4.39E-05 |
|  |  |  | A0A6P5RYK4 | -1.260399601 | 4.40E-05 |
|  |  |  | A0A6P5SAH1 | -0.585392344 | 4.44E-05 |
|  |  |  | A0A6P5S023 | -1.205330012 | 4.46E-05 |
|  |  |  | A0A6P5T7A6 | -0.592022118 | 4.56E-05 |
|  |  |  | A0A6P5SWP2 | -0.738314934 | 4.60E-05 |
|  |  |  | A0A6P5TCE0 | -1.594718924 | 4.64E-05 |
|  |  |  | A0A6P5RIK6 | -0.980338041 | 4.66E-05 |
|  |  |  | A0A6P5R662 | -0.848389591 | 4.67E-05 |
|  |  |  | A0A6P5SZP9 | -0.585509454 | 4.74E-05 |
|  |  |  | A0A6P5U0Q8 | -0.853765373 | 4.79E-05 |
|  |  |  | A0A6P5T8K5 | -0.59044662 | 4.82E-05 |
|  |  |  | A0A6P5SI65 | -2.965272412 | 4.82E-05 |
|  |  |  | A0A6P5T948 | -0.888589235 | 4.90E-05 |
|  |  |  | A0A6P5SB11 | -0.82159134 | 5.05E-05 |
|  |  |  | A0A6P5RW08 | -0.629519113 | 5.12E-05 |
|  |  |  | A0A6P5S5I2 | -0.7015379 | 5.14E-05 |
|  |  |  | A0A6P5U3E9 | -0.669352019 | 5.18E-05 |
|  |  |  | A0A6P5SZV0 | -0.992557532 | 5.20E-05 |
|  |  |  | A0A6P5TNE7 | -0.740962396 | 5.21E-05 |
|  |  |  | A0A6P5SJ40 | -0.694135733 | 5.22E-05 |
|  |  |  | A0A6P5TH86 | -1.369549708 | 5.25E-05 |
|  |  |  | A0A6P5T1Z6 | -0.722738731 | 5.35E-05 |
|  |  |  | A0A6P5RDD2 | -1.442891863 | 5.40E-05 |
|  |  |  | A0A6P5SGI4 | -0.723840593 | 5.46E-05 |
|  |  |  | A0A6P5U437 | -1.63863998 | 5.62E-05 |
|  |  |  | A0A6P5T963 | -0.772077318 | 5.66E-05 |
|  |  |  | A0A6P5SFH0 | -1.479416031 | 5.71E-05 |
|  |  |  | A0A6P5SRD7 | -0.609842847 | 5.75E-05 |
|  |  |  | A0A6P5RYB3 | -0.731624828 | 5.86E-05 |
|  |  |  | A0A6P5TEI1 | -0.605384273 | 5.95E-05 |
|  |  |  | A0A6P5S118 | -0.639280224 | 6.00E-05 |
|  |  |  | A0A6P5RHB1 | -0.801256735 | 6.16E-05 |
|  |  |  | A0A6P5S300 | -0.64320218 | 6.39E-05 |
|  |  |  | A0A6P5TYJ2 | -0.872969247 | 6.40E-05 |
|  |  |  | A0A6P5RKT8 | -0.658459981 | 6.44E-05 |
|  |  |  | A0A6P5STQ4 | -0.68737629 | 6.60E-05 |
|  |  |  | A0A6P5TKV6 | -0.987223571 | 6.80E-05 |
|  |  |  | A0A6P5TBJ5 | -1.122075825 | 7.23E-05 |
|  |  |  | A0A6P5SHP0 | -0.694748081 | 7.36E-05 |
|  |  |  | A0A6P5RZL0 | -0.773937716 | 7.37E-05 |
|  |  |  | A0A6P5TJ40 | -0.621435567 | 7.47E-05 |
|  |  |  | A0A6P5U3M0 | -0.718587895 | 7.54E-05 |
|  |  |  | A0A6P5RUA7 | -0.845196492 | 7.92E-05 |
|  |  |  | A0A6P5RGN9 | -1.109865982 | 7.97E-05 |
|  |  |  | A0A6P5SSU7 | -0.665957001 | 8.25E-05 |
|  |  |  | A0A6P5RJ96 | -0.692980287 | 8.55E-05 |
|  |  |  | A0A6P5SRR8 | -0.610623261 | 8.66E-05 |
|  |  |  | A0A6P5RZY0 | -0.934571861 | 8.75E-05 |
|  |  |  | A0A6P5TXU8 | -0.786955278 | 8.80E-05 |
|  |  |  | A0A6P5SEI8 | -1.284908807 | 8.85E-05 |
|  |  |  | A0A6P5S4Y6 | -0.7188224 | 9.63E-05 |
|  |  |  | A0A6P5SGR6 | -0.80402982 | 0.000102959 |
|  |  |  | A0A6P5S4L3 | -0.660950753 | 0.000105195 |
|  |  |  | A0A6P5S845 | -0.779909699 | 0.00010828 |
|  |  |  | A0A6P5RKX6 | -0.948972412 | 0.00010946 |
|  |  |  | A0A6P5SLG9 | -0.826740227 | 0.000110586 |
|  |  |  | A0A6P5SN30 | -1.024474607 | 0.000114636 |
|  |  |  | A0A6P5RPM8 | -0.608802258 | 0.000115819 |
|  |  |  | A0A6P5S1R5 | -0.716125745 | 0.000117348 |
|  |  |  | A0A6P5RZ42 | -0.636025099 | 0.000117901 |
|  |  |  | A0A6P5SIS0 | -1.242789857 | 0.000119073 |
|  |  |  | A0A6P5TY24 | -0.966668522 | 0.000120242 |
|  |  |  | A0A6P5SIB8 | -0.680818884 | 0.000121766 |
|  |  |  | A0A6P5SWD4 | -0.776238779 | 0.000123013 |
|  |  |  | A0A6P5SPT7 | -0.865884922 | 0.000124362 |
|  |  |  | A0A6P5S955 | -0.656515184 | 0.000128673 |
|  |  |  | A0A6P5TUV2 | -1.062967428 | 0.000131588 |
|  |  |  | A0A6P5RYZ7 | -0.686722689 | 0.000136066 |
|  |  |  | A0A6P5SWX7 | -0.595366177 | 0.000158348 |
|  |  |  | A0A6P5THH5 | -1.081237575 | 0.000160409 |
|  |  |  | A0A6P5TFE2 | -0.731291576 | 0.000163385 |
|  |  |  | A0A6P5TEI0 | -0.608931682 | 0.000169375 |
|  |  |  | A0A6P5TEB2 | -0.685330199 | 0.000170533 |
|  |  |  | A0A6P5SNV3 | -0.788764158 | 0.000174778 |
|  |  |  | A0A6P5TNT7 | -0.797217688 | 0.000185114 |
|  |  |  | A0A6P5TY28 | -0.969318045 | 0.00018831 |
|  |  |  | A0A6P5THS0 | -0.62588779 | 0.000194688 |
|  |  |  | A0A6P5TCI6 | -0.797713772 | 0.00019934 |
|  |  |  | A0A6P5SRQ1 | -1.290629813 | 0.000199403 |
|  |  |  | A0A6P5SZF5 | -0.604668742 | 0.000211062 |
|  |  |  | A0A6P5SFF0 | -0.996064752 | 0.000222984 |
|  |  |  | A0A6P5SFX0 | -0.819554251 | 0.000228899 |
|  |  |  | A0A6P5SJD4 | -1.341394341 | 0.00023333 |
|  |  |  | A0A6P5TCK4 | -1.264240087 | 0.000233569 |
|  |  |  | A0A6P5S1B0 | -0.621112515 | 0.000237817 |
|  |  |  | A0A6P5SDM4 | -1.496291425 | 0.000241834 |
|  |  |  | A0A6P5SMW9 | -0.764353052 | 0.000246309 |
|  |  |  | A0A6P5S4L5 | -0.768474213 | 0.000251893 |
|  |  |  | A0A6P5S3L5 | -1.3154385 | 0.000257145 |
|  |  |  | A0A6P5U5N3 | -0.930545569 | 0.000261885 |
|  |  |  | A0A6P5TC31 | -1.005445583 | 0.000268575 |
|  |  |  | A0A6P5T8I7 | -1.025451334 | 0.000268601 |
|  |  |  | A0A6P5TLM9 | -0.613714472 | 0.000279649 |
|  |  |  | A0A6P5TJ42 | -0.665857773 | 0.000282955 |
|  |  |  | A0A6P5T7I7 | -0.641621807 | 0.000283839 |
|  |  |  | A0A6P5U5L0 | -0.821700384 | 0.000284388 |
|  |  |  | A0A6P5TZ40 | -0.638418871 | 0.000284897 |
|  |  |  | A0A6P5RS14 | -1.102296965 | 0.000406596 |
|  |  |  | A0A6P5RDS7 | -0.813022921 | 0.000298058 |
|  |  |  | A0A6P5T053 | -1.129565171 | 0.000304822 |
|  |  |  | A0A6P5RLR5 | -1.046781398 | 0.00031608 |
|  |  |  | A0A6P5RVZ1 | -1.082007279 | 0.000333372 |
|  |  |  | A0A6P5TC35 | -0.740781464 | 0.000345872 |
|  |  |  | A0A6P5SJB8 | -0.959314411 | 0.000350912 |
|  |  |  | A0A6P5S487 | -0.70457965 | 0.000362615 |
|  |  |  | A0A6P5SAB2 | -0.640931609 | 0.000366976 |
|  |  |  | A0A6P5SBM9 | -0.656243224 | 0.000392452 |
|  |  |  | A0A6P5TZC5 | -1.003169087 | 0.000392626 |
|  |  |  | A0A6P5SIE4 | -0.64653488 | 0.000411678 |
|  |  |  | A0A6P5TIT9 | -0.613032582 | 0.000419225 |
|  |  |  | A0A6P5RIC0 | -0.617856954 | 0.000432432 |
|  |  |  | A0A6P5T043 | -0.715086852 | 0.000473428 |
|  |  |  | A0A6P5TPN6 | -0.596001686 | 0.00049757 |
|  |  |  | A0A6P5TCY8 | -1.443897754 | 0.000553804 |
|  |  |  | A0A6P5SG61 | -0.802128123 | 0.000571935 |
|  |  |  | A0A6P5T8K7 | -1.054882709 | 0.000579206 |
|  |  |  | A0A6P5S9V7 | -0.73832989 | 0.000582536 |
|  |  |  | A0A6P5T5S9 | -0.783185448 | 0.000605776 |
|  |  |  | A0A6P5TIF4 | -0.759659572 | 0.000649449 |
|  |  |  | A0A6P5TEM1 | -0.585267622 | 0.000654868 |
|  |  |  | A0A6P5S8T1 | -0.870764177 | 0.000658024 |
|  |  |  | A0A6P5S1E5 | -0.831992297 | 0.000664424 |
|  |  |  | A0A6P5SH32 | -0.626328246 | 0.000692009 |
|  |  |  | A0A6P5TWN6 | -1.028965857 | 0.000746395 |
|  |  |  | A0A6P5U2R9 | -0.822495615 | 0.000747433 |
|  |  |  | A0A6P5U362 | -0.670026408 | 0.000755682 |
|  |  |  | A0A6P5TJ73 | -0.590292918 | 0.000799845 |
|  |  |  | A0A6P5TSV5 | -1.422323546 | 0.000850516 |
|  |  |  | A0A6P5RB25 | -1.197894964 | 0.00086214 |
|  |  |  | A0A6P5SDC5 | -0.811779543 | 0.00117262 |
|  |  |  | A0A6P5SS98 | -0.769611691 | 0.000880274 |
|  |  |  | A0A6P5TM74 | -1.459985616 | 0.000933167 |
|  |  |  | A0A6P5S880 | -0.606682663 | 0.000983429 |
|  |  |  | A0A6P5R736 | -0.650064826 | 0.001001035 |
|  |  |  | A0A6P5TGX3 | -0.818884381 | 0.001056991 |
|  |  |  | A0A6P5THX5 | -0.672707792 | 0.001069105 |
|  |  |  | A0A6P5RL02 | -1.015431663 | 0.00109361 |
|  |  |  | A0A6P5REW7 | -0.595692518 | 0.001121815 |
|  |  |  | A0A6P5S7Y2 | -0.704556797 | 0.00123943 |
|  |  |  | A0A6P5THD6 | -1.392171394 | 0.001282079 |
|  |  |  | A0A6P5SFS4 | -0.683116301 | 0.001359621 |
|  |  |  | A0A6P5T587 | -0.735357794 | 0.001379403 |
|  |  |  | A0A6P5TFX8 | -0.745026852 | 0.001474014 |
|  |  |  | A0A6P5SJB4 | -1.349385505 | 0.001582771 |
|  |  |  | A0A6P5SKA4 | -1.034187472 | 0.001630326 |
|  |  |  | A0A6P5T004 | -1.460805581 | 0.001685368 |
|  |  |  | A0A6P5T6C4 | -0.910271876 | 0.001736772 |
|  |  |  | A0A6P5TS82 | -0.803557647 | 0.001766128 |
|  |  |  | A0A6P5TXS3 | -0.734704821 | 0.001859211 |
|  |  |  | A0A6P5RRI7 | -0.721736841 | 0.001896759 |
|  |  |  | A0A6P5RFJ5 | -0.605772749 | 0.002508731 |
|  |  |  | A0A6P5S0J3 | -1.54892354 | 0.001906249 |
|  |  |  | A0A6P5SPJ9 | -1.023371896 | 0.001909588 |
|  |  |  | A0A6P5T667 | -0.818020509 | 0.001981718 |
|  |  |  | A0A6P5TRS0 | -2.385942716 | 0.002689026 |
|  |  |  | A0A6P5RS13 | -0.755249197 | 0.002040863 |
|  |  |  | A0A6P5TWD0 | -1.612331563 | 0.00229864 |
|  |  |  | A0A6P5SUX0 | -0.726459366 | 0.002311603 |
|  |  |  | A0A6P5RVE4 | -0.681403099 | 0.00246773 |
|  |  |  | A0A6P5TME5 | -0.768725712 | 0.002497545 |
|  |  |  | A0A6P5RMQ1 | -0.815201126 | 0.00257602 |
|  |  |  | A0A6P5RTM7 | -0.893399693 | 0.002722369 |
|  |  |  | A0A6P5RVG2 | -0.688857881 | 0.002728223 |
|  |  |  | A0A6P5T6N7 | -0.798042161 | 0.002785691 |
|  |  |  | A0A6P5S4U6 | -0.924346575 | 0.002867335 |
|  |  |  | A0A6P5RUN8 | -0.674398995 | 0.002925225 |
|  |  |  | A0A6P5SBD1 | -0.69632636 | 0.00371582 |
|  |  |  | A0A6P5RBL7 | -1.603991264 | 0.004011275 |
|  |  |  | A0A6P5RS82 | -0.645304731 | 0.004183472 |
|  |  |  | A0A6P5SW32 | -0.625874263 | 0.004207742 |
|  |  |  | A0A6P5TB97 | -0.753331478 | 0.004214025 |
|  |  |  | A0A6P5TMD9 | -0.683391098 | 0.004427877 |
|  |  |  | A0A6P5TX24 | -0.735333308 | 0.004541124 |
|  |  |  | A0A6P5SC61 | -0.775526569 | 0.004776328 |
|  |  |  | A0A6P5T9K2 | -0.739303209 | 0.005402639 |
|  |  |  | A0A6P5RW06 | -0.737548131 | 0.005789293 |
|  |  |  | A0A6P5TWZ1 | -0.72365547 | 0.006078785 |
|  |  |  | A0A6P5SD79 | -1.745730779 | 0.006783164 |
|  |  |  | A0A6P5T6L7 | -0.810490908 | 0.006847878 |
|  |  |  | A0A6P5SGV0 | -0.592783286 | 0.006993815 |
|  |  |  | A0A6P5TTE8 | -0.711208557 | 0.007168587 |
|  |  |  | A0A6P5T6C3 | -0.744017742 | 0.007850477 |
|  |  |  | A0A6P5TBP6 | -1.713263782 | 0.008472727 |
|  |  |  | A0A6P5TQE8 | -1.446281743 | 0.008704946 |
|  |  |  | A0A6P5RZP5 | -1.123972693 | 0.010871415 |
|  |  |  | A0A6P5SUY0 | -0.604069009 | 0.011299539 |
|  |  |  | A0A6P5U1P6 | -0.922113343 | 0.012388954 |
|  |  |  | A0A6P5RYB2 | -0.978133364 | 0.013628608 |
|  |  |  | A0A6P5TXY8 | -0.709160736 | 0.01588353 |
|  |  |  | A0A6P5TPF4 | -0.587586382 | 0.016436405 |
|  |  |  | A0A6P5RRQ9 | -0.615169064 | 0.017890796 |
|  |  |  | A0A6P5TS94 | -0.596846344 | 0.018698776 |
|  |  |  | A0A6P5SCI9 | -0.623586332 | 0.019836432 |
|  |  |  | A0A6P5TNQ5 | -0.595816205 | 0.020697417 |
|  |  |  | A0A6P5TB31 | -1.04905775 | 0.020888856 |
|  |  |  | A0A6P5SKK6 | -1.128023096 | 0.023006025 |
|  |  |  | A0A6P5S633 | -0.651031982 | 0.028117145 |
|  |  |  | A0A6P5T9P0 | -0.609880748 | 0.031751703 |


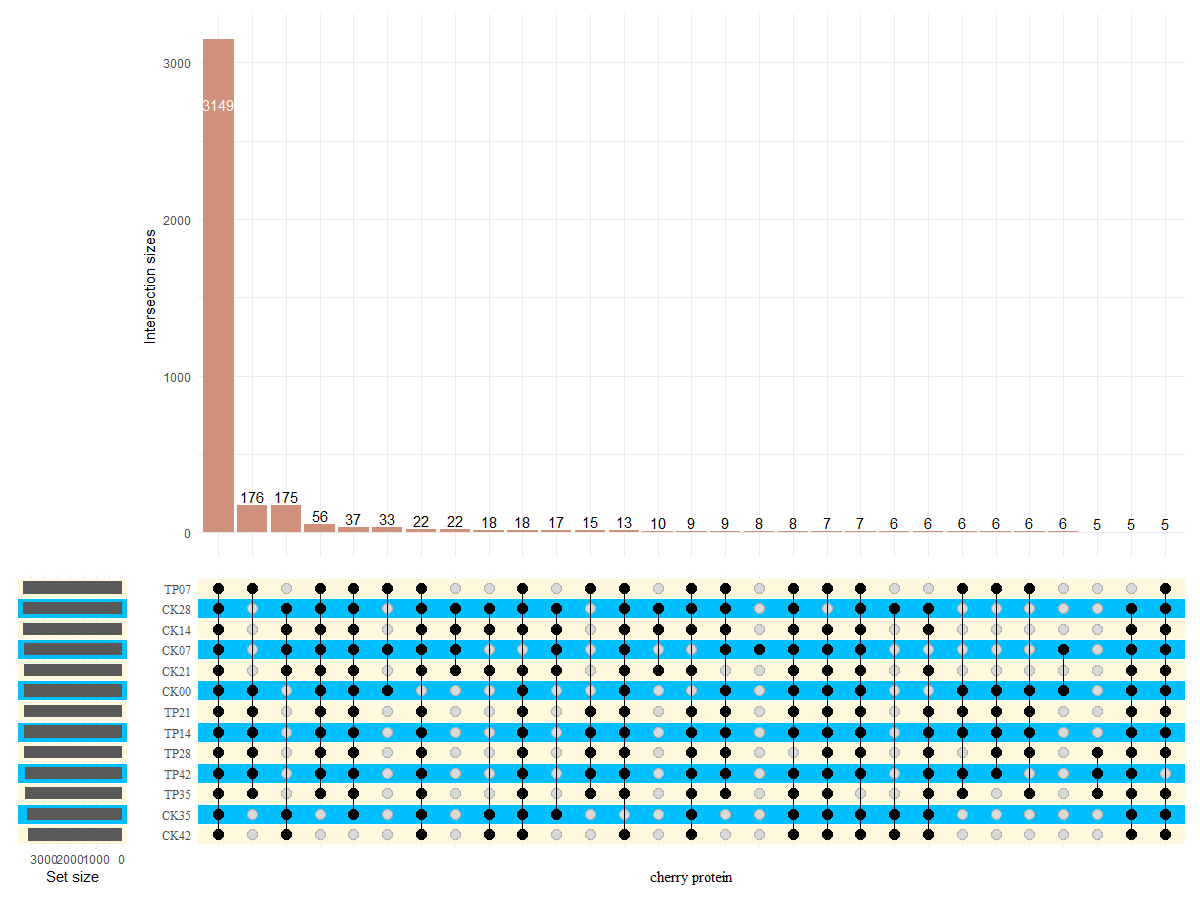


**Figure S1** Upset diagram of the number of different proteins identified in the cherry fruit proteome in 13 samples.

| 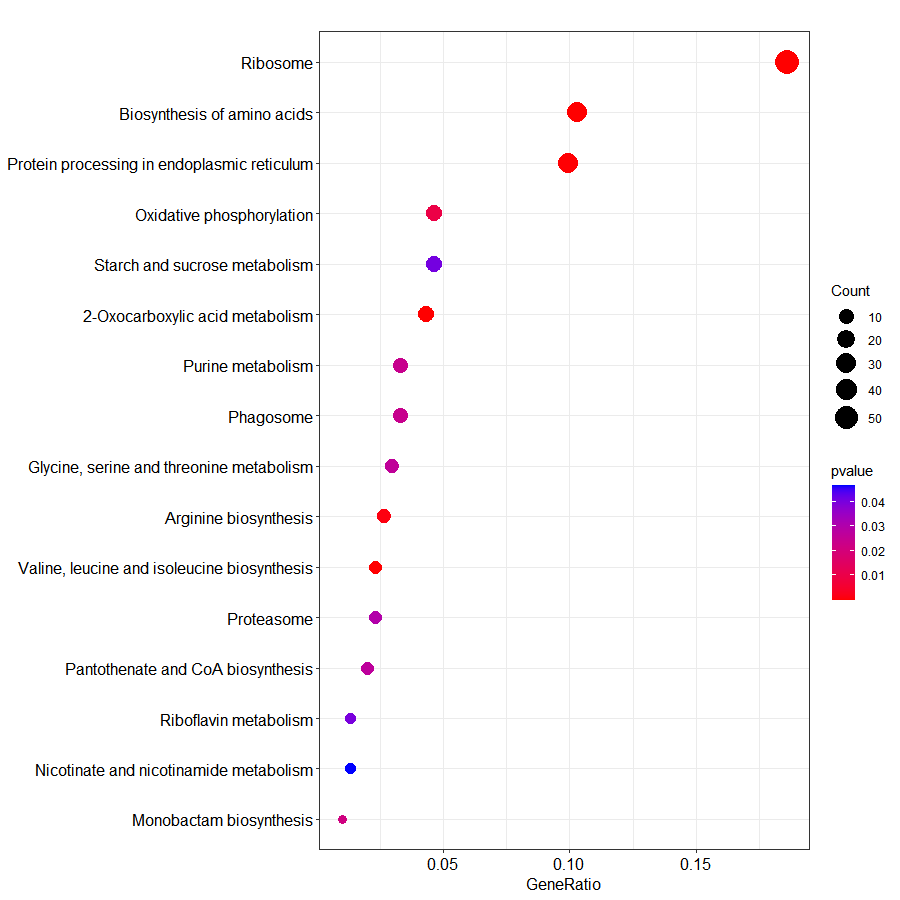 | 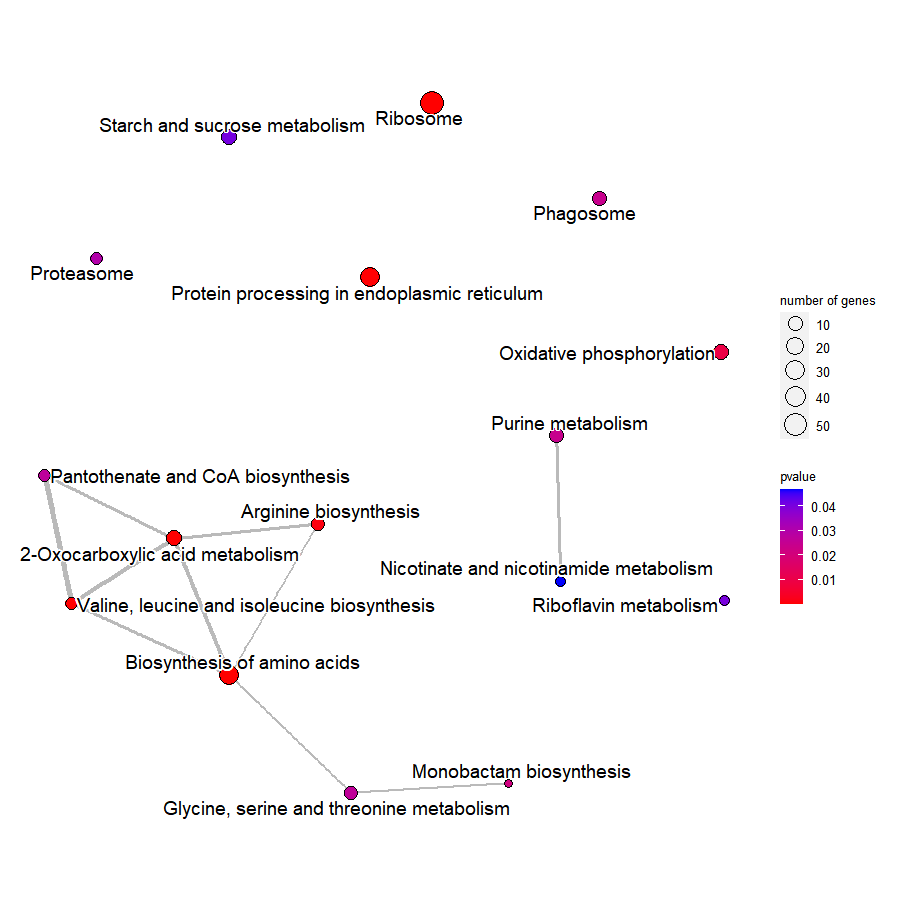 |
| --- | --- |
| (a)KEGG and emapplot of group 1 | |
| 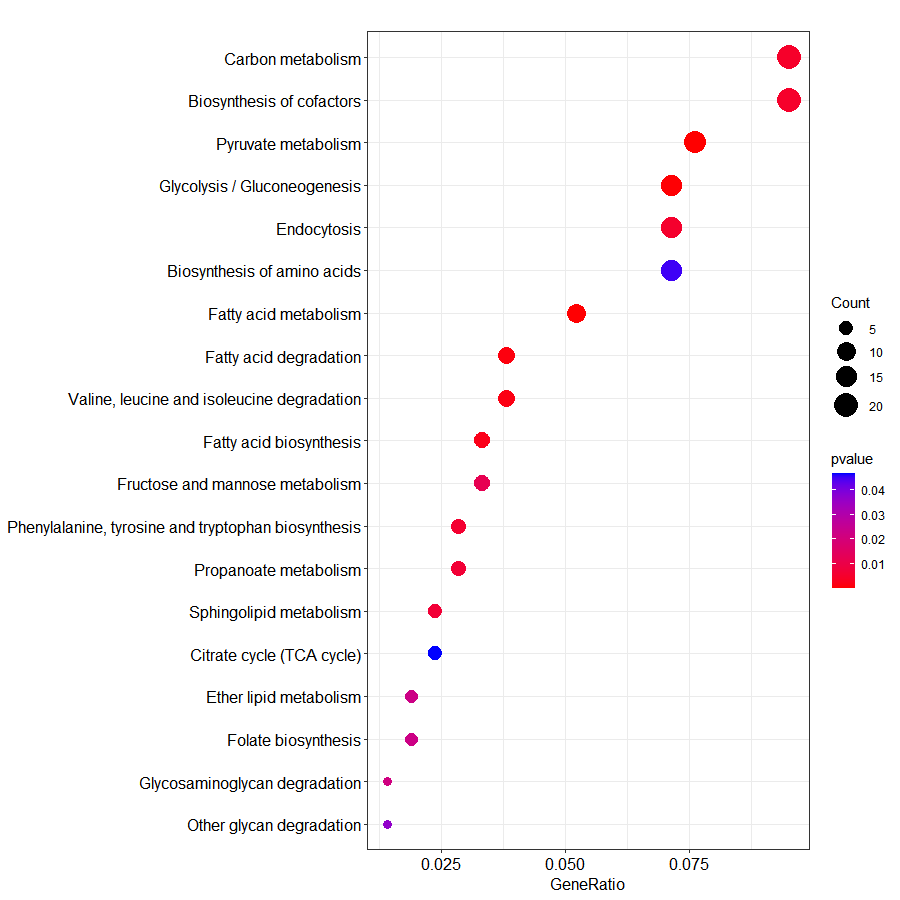 | 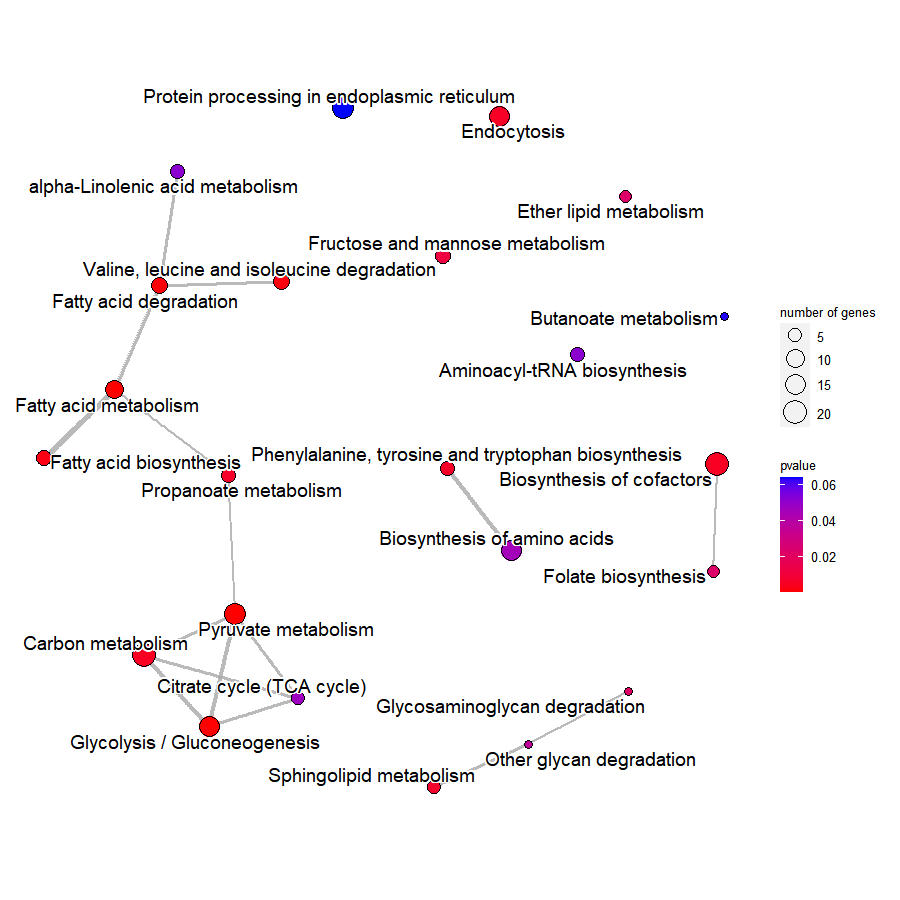 |
| (b)KEGG and emapplot of group 2 | |
| 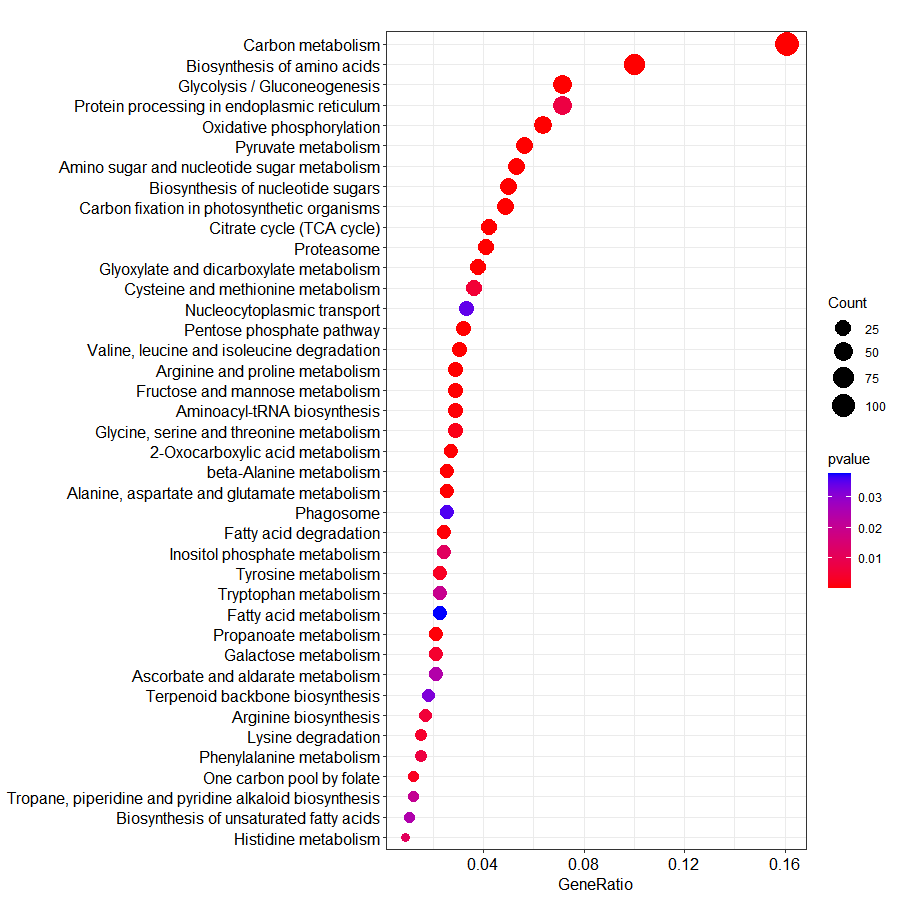 | 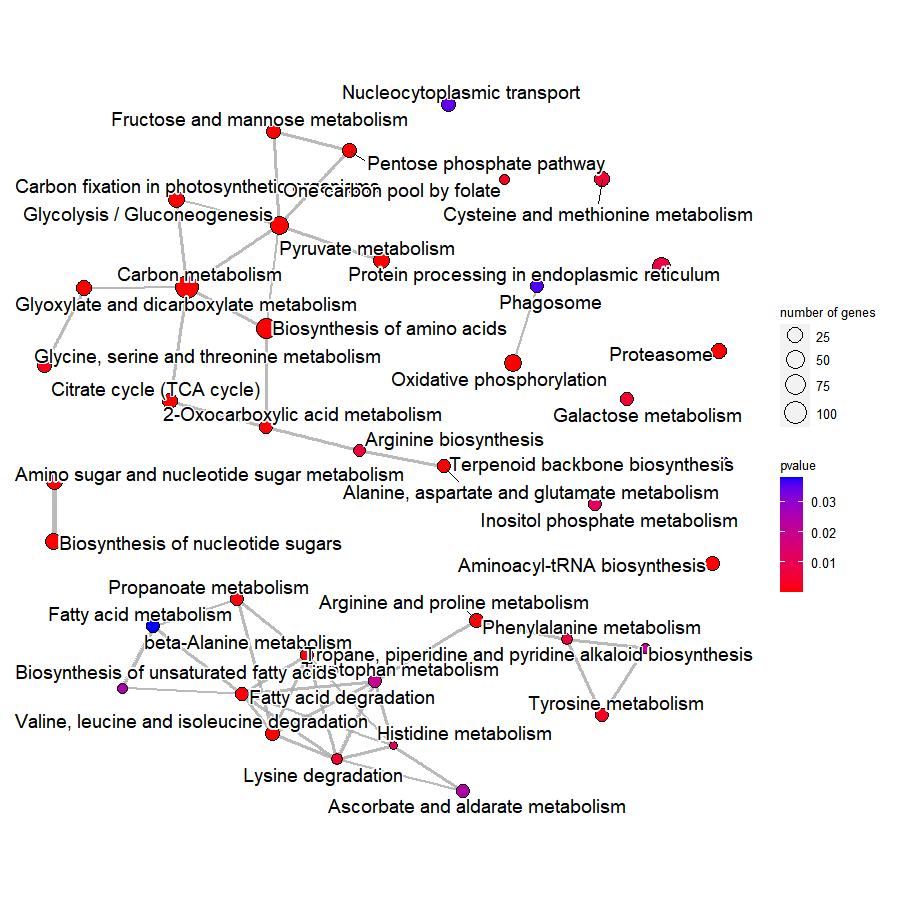 |
| (c)KEGG and emapplot of group 3 | |
| 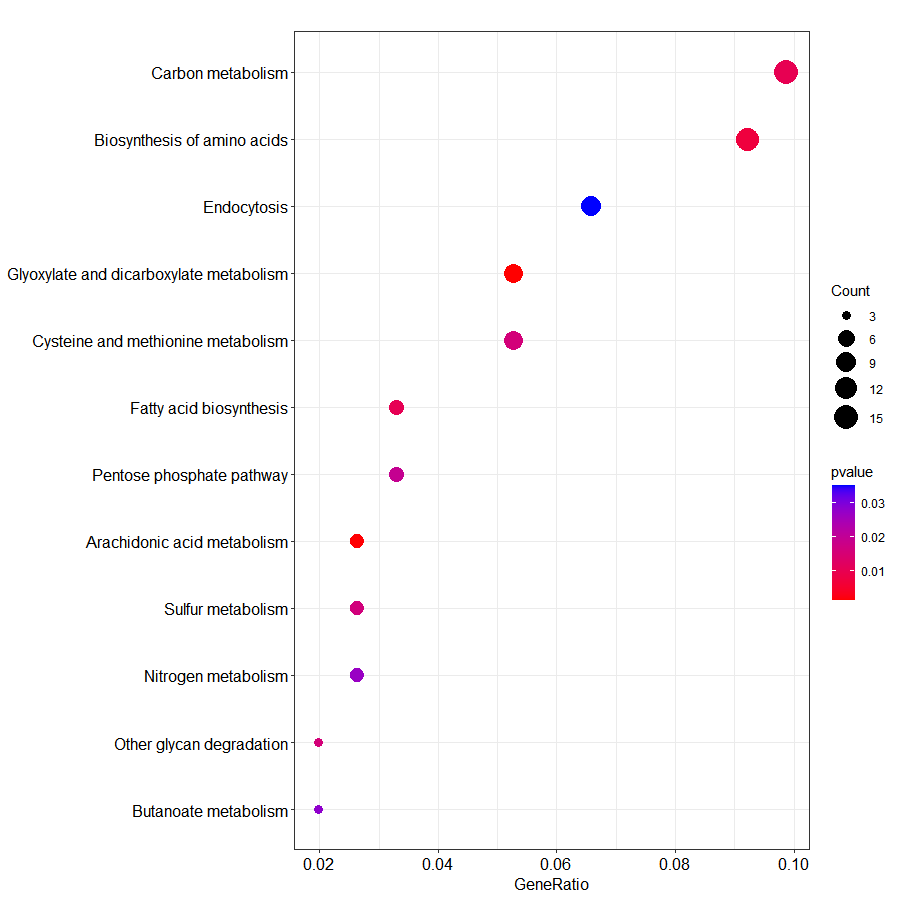 | 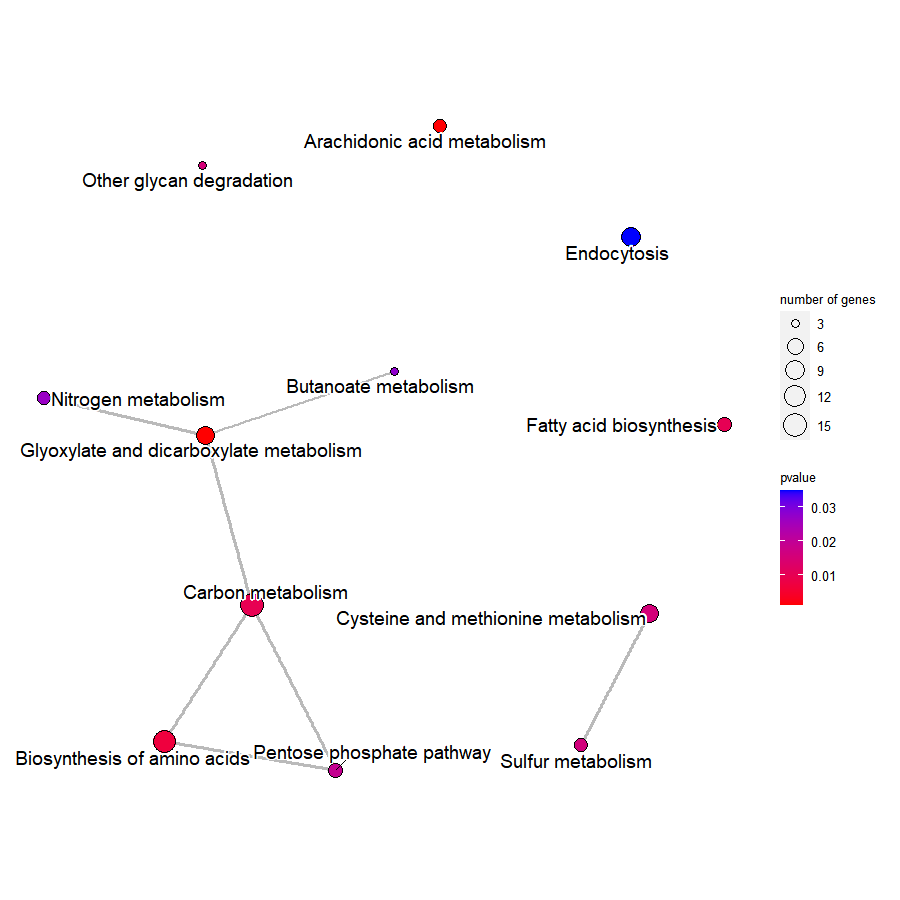 |
| (d)KEGG and emapplot of group 4 | |
| **FIGURE S2** KEGG and emapplot of four groups | |

**FIGURE S3** KEGG pathways of DEPs in group3 (**FIGURE 6**)

| 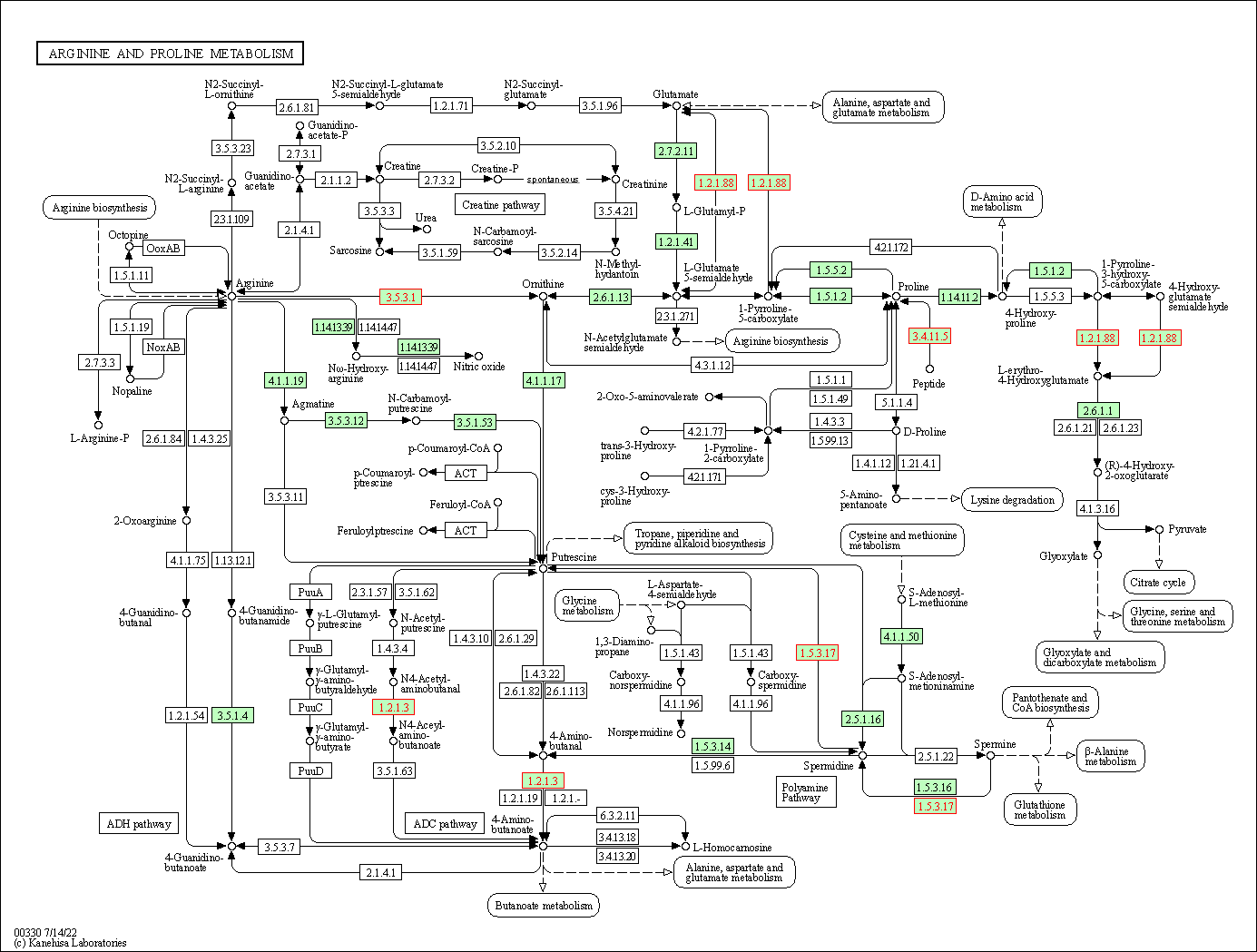 | 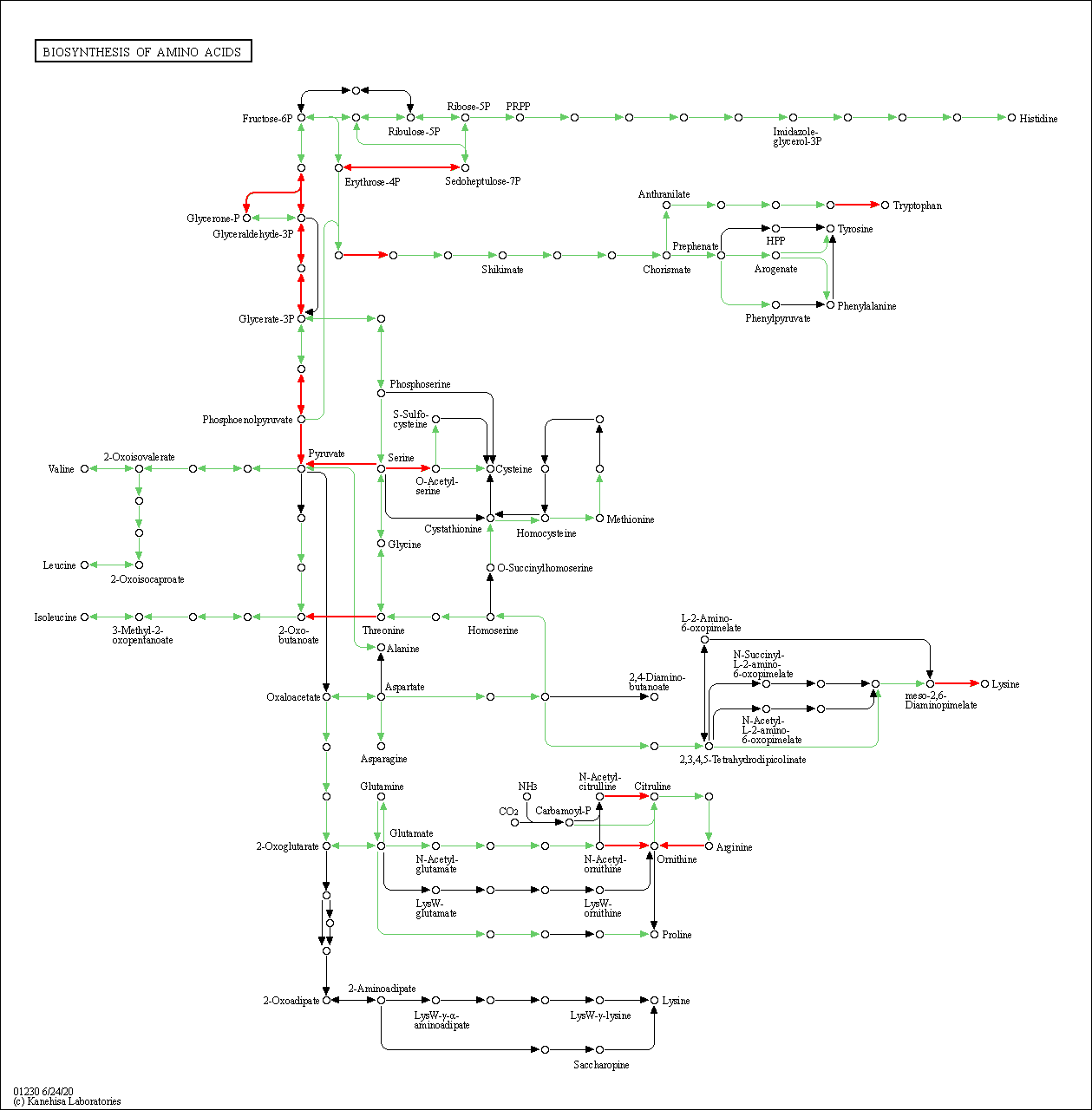 |
| --- | --- |
| 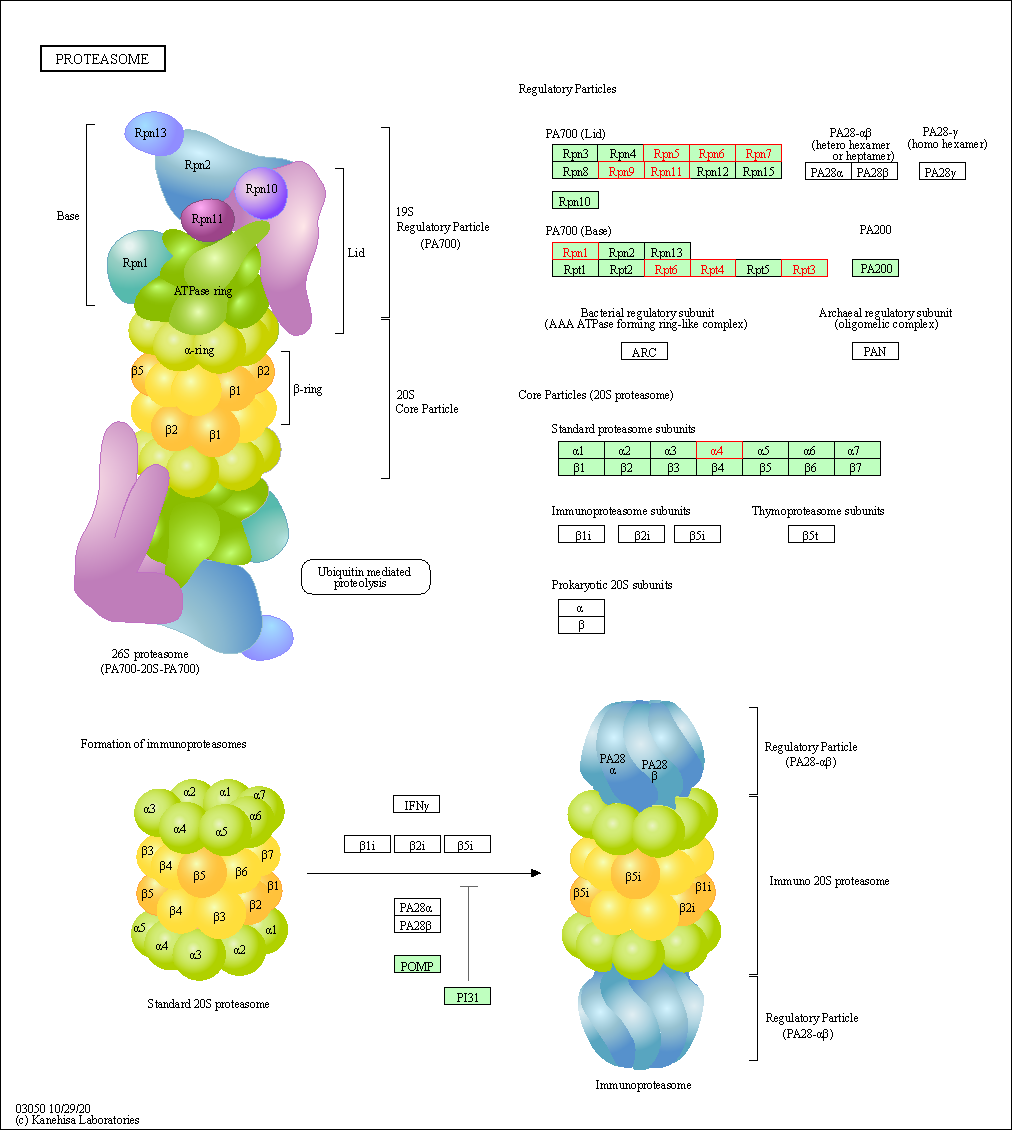 | 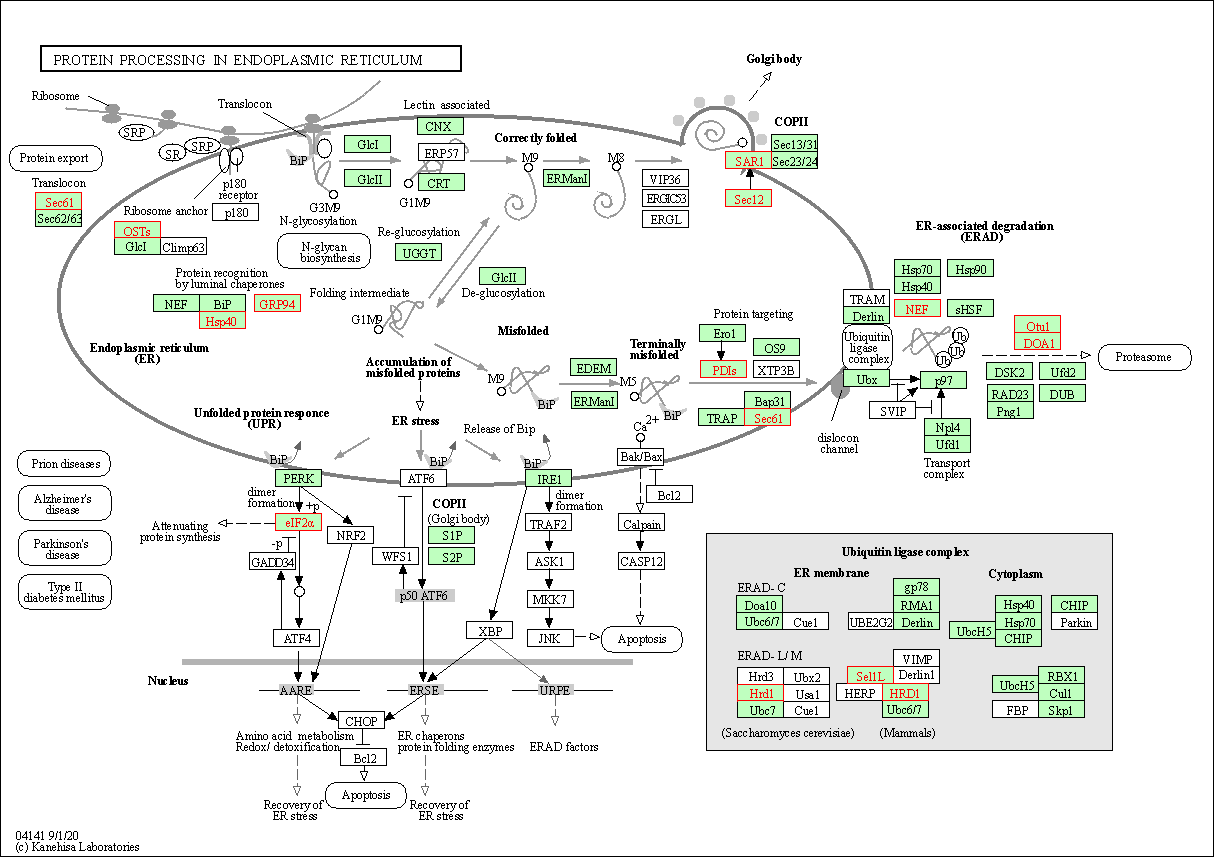 |
| 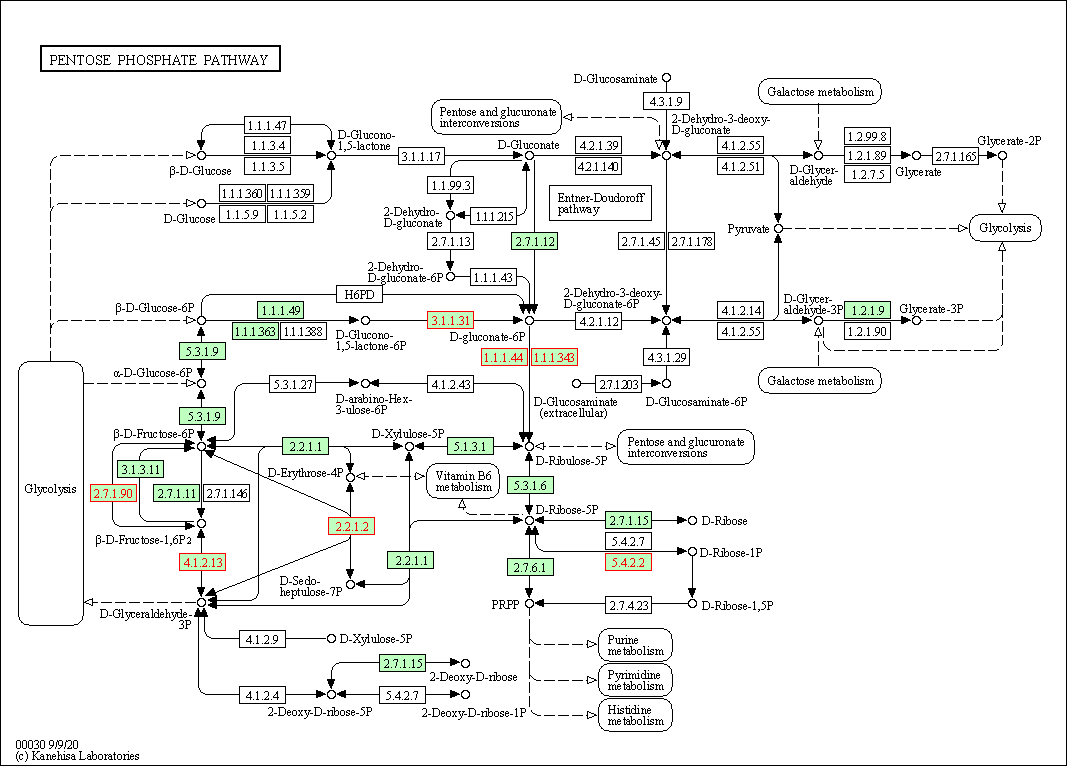 | 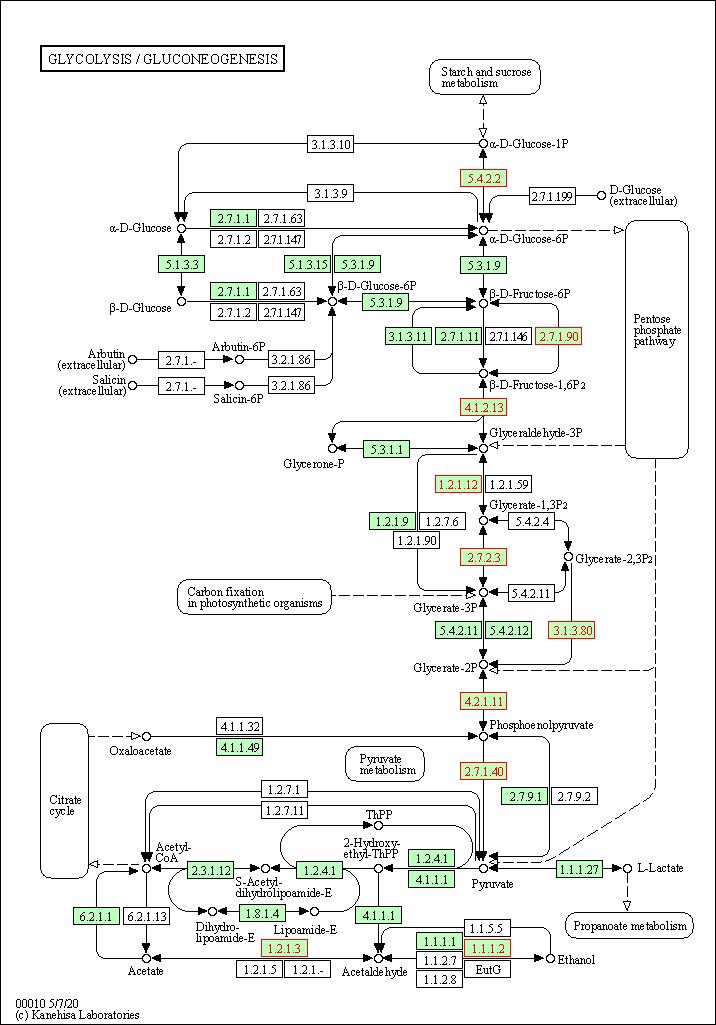 |
| 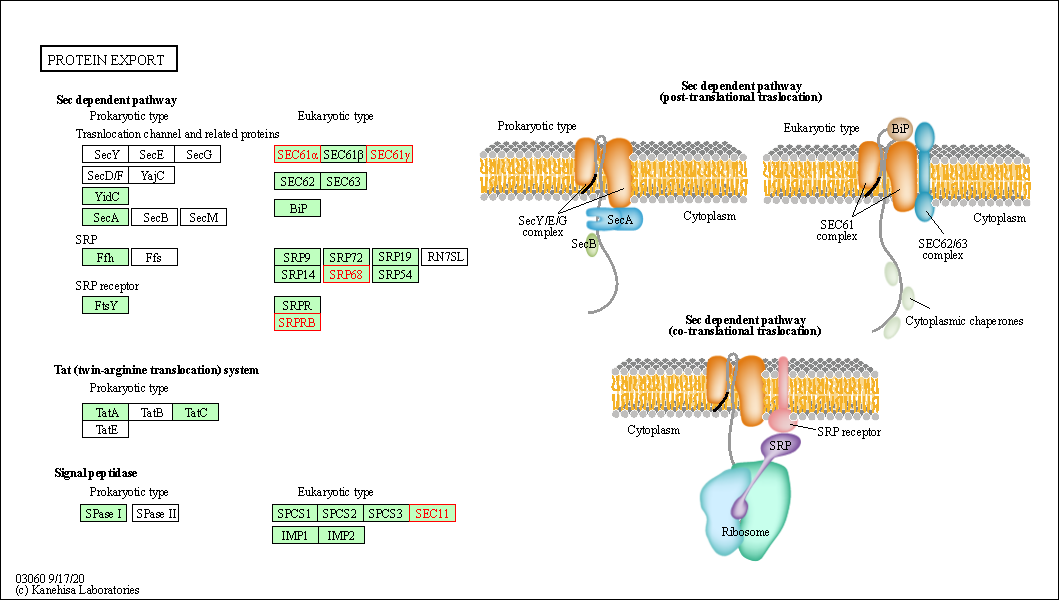 | 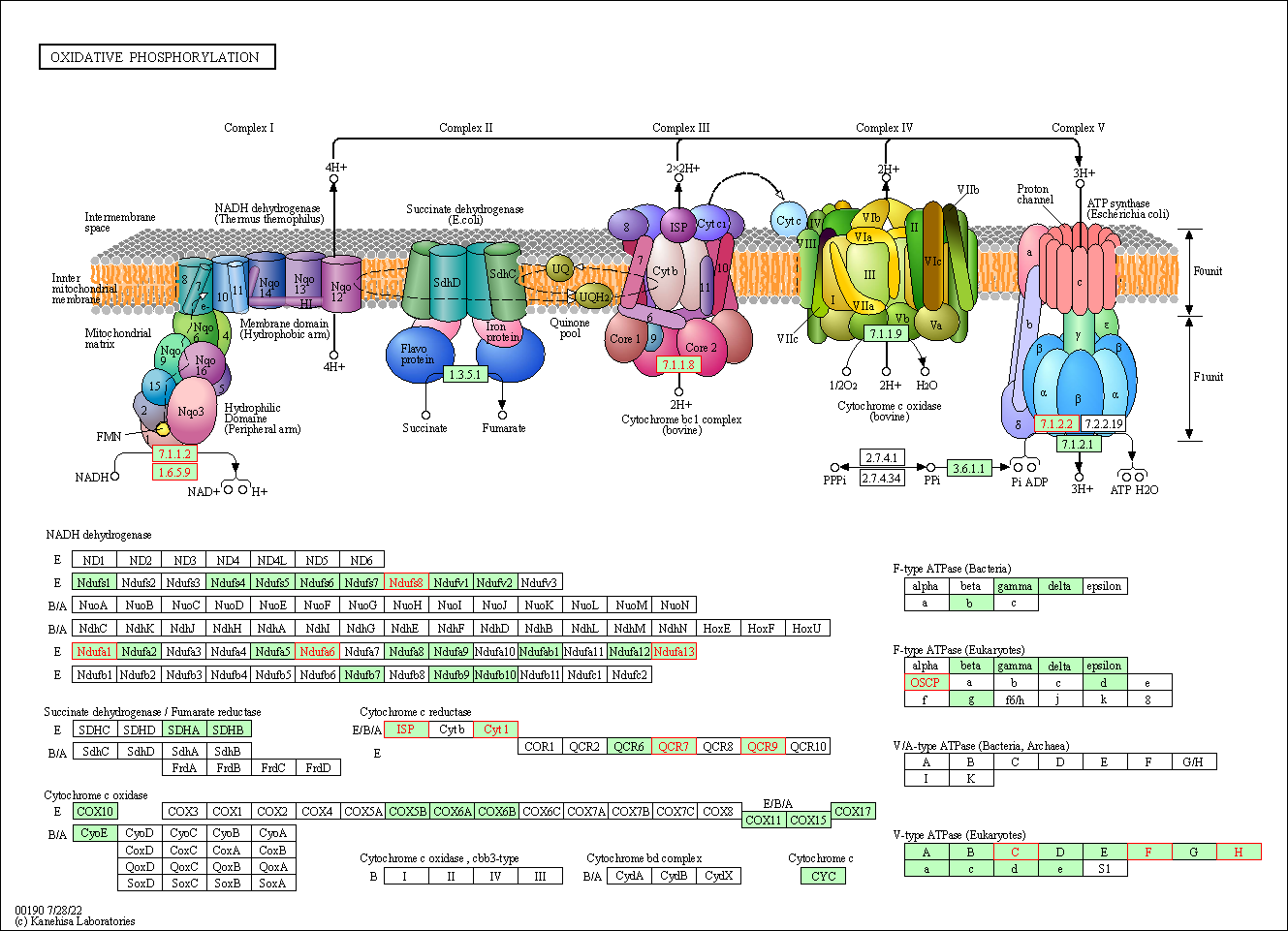 |
| 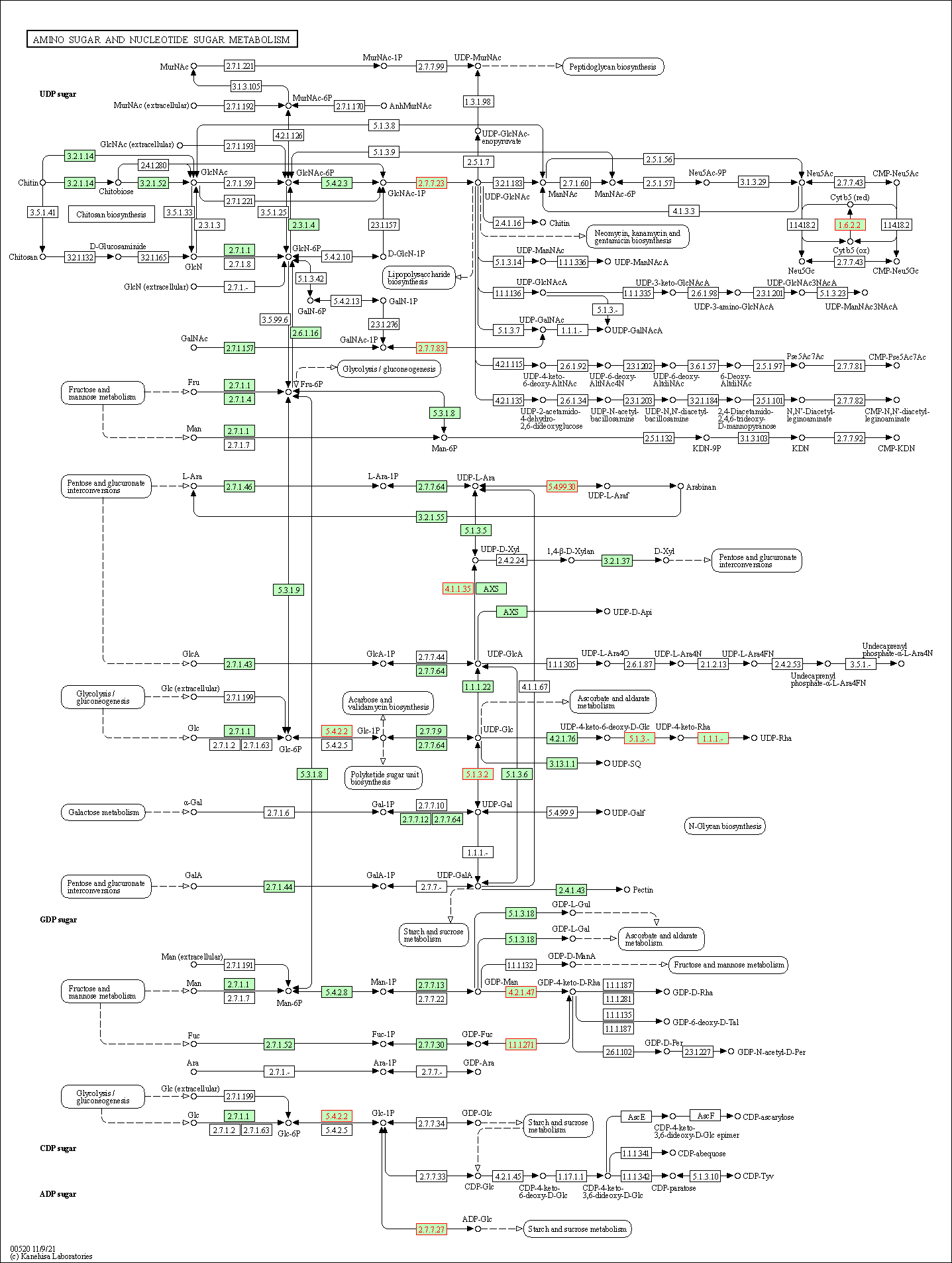 | 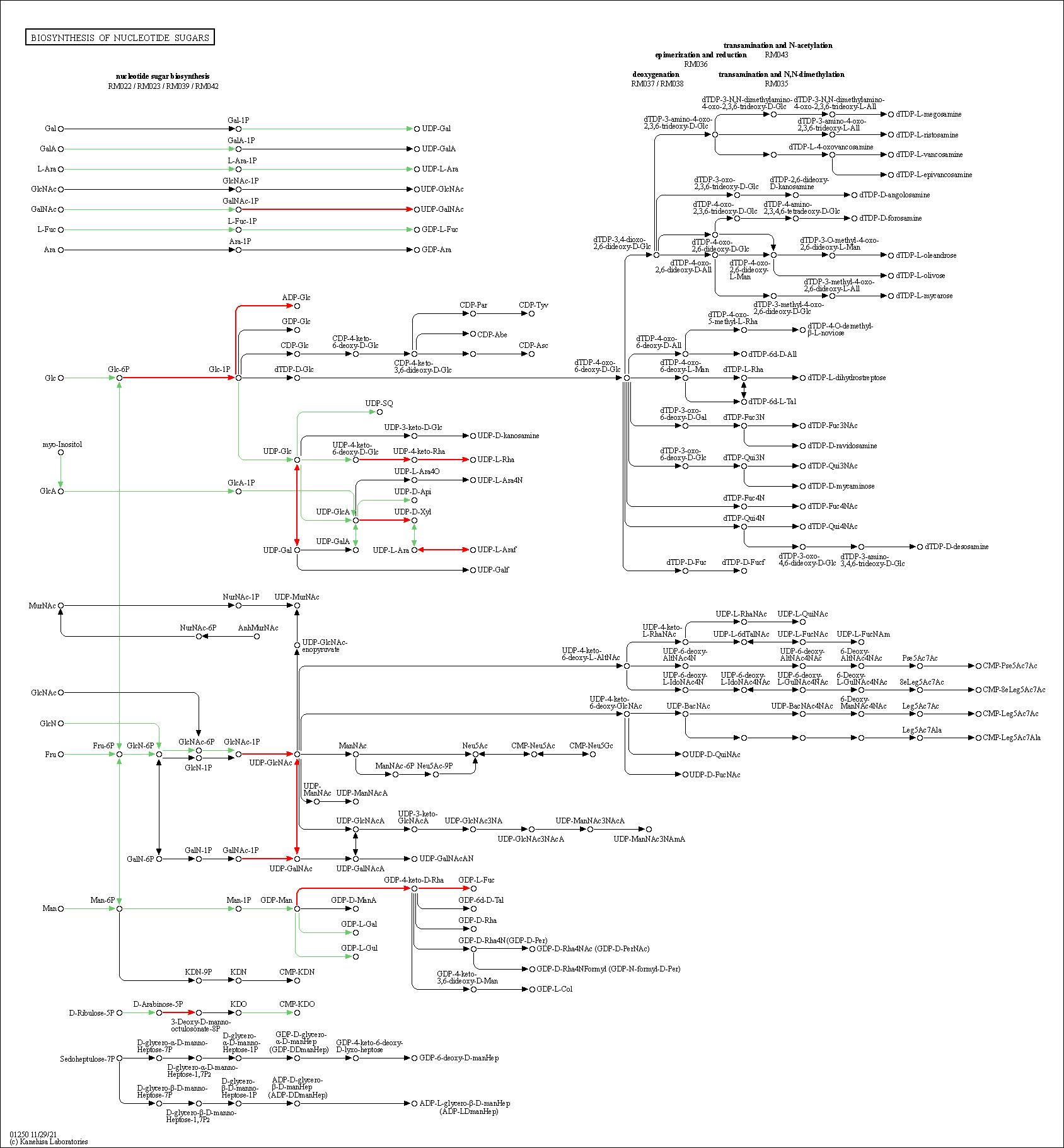 |
| 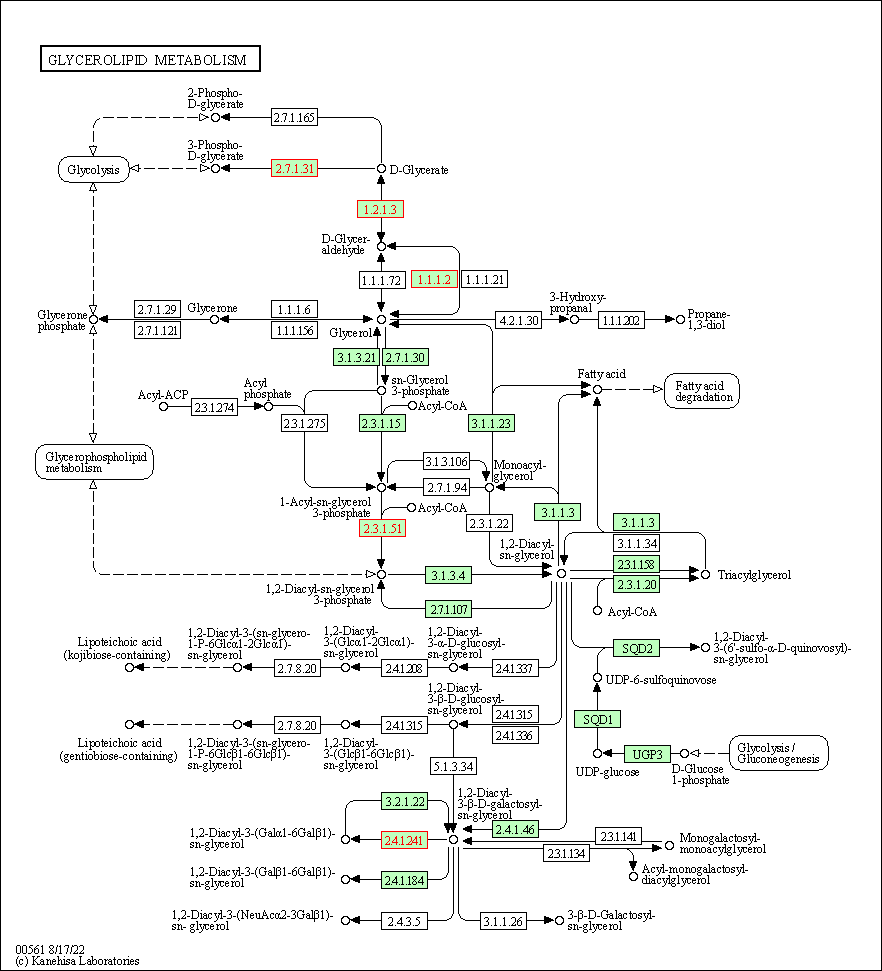 | 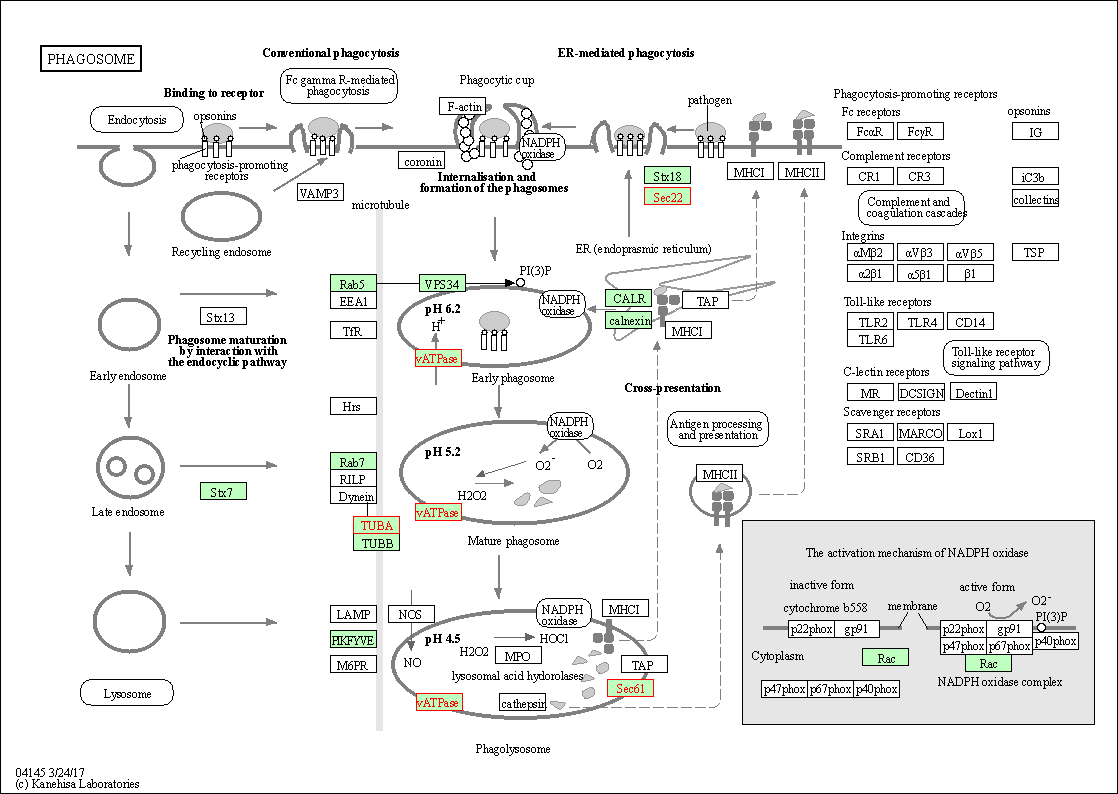 |
| 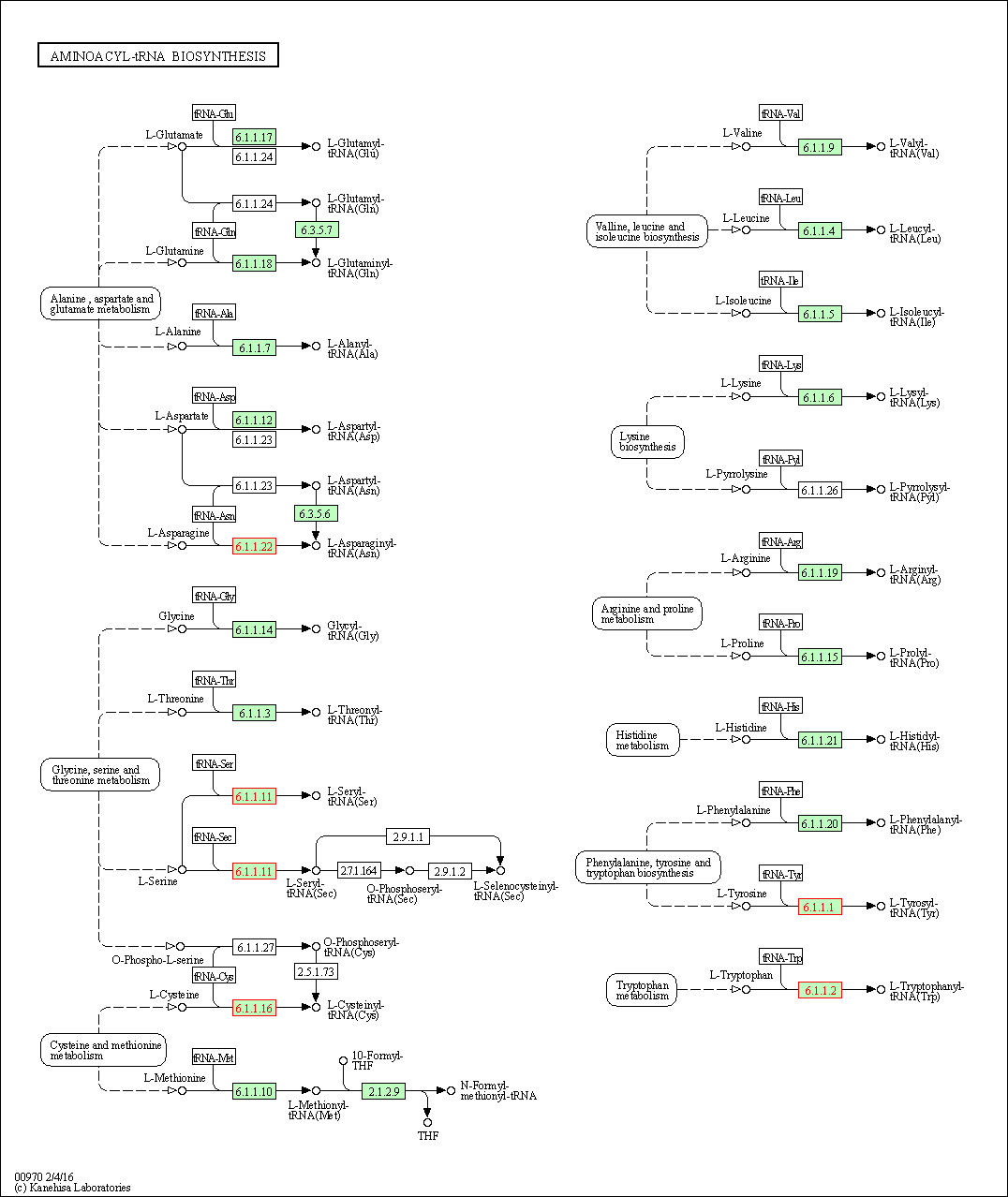 | 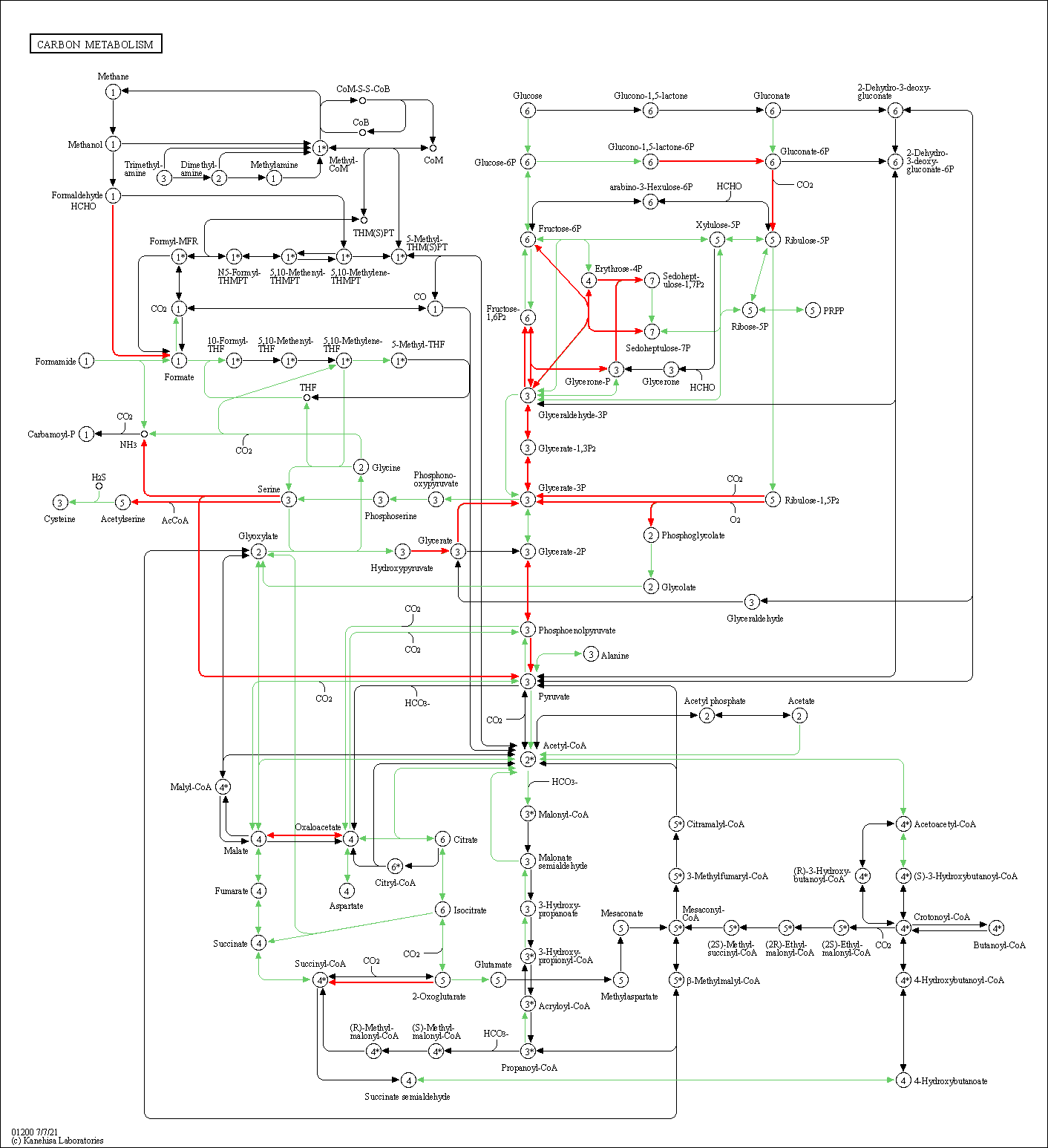 |
| 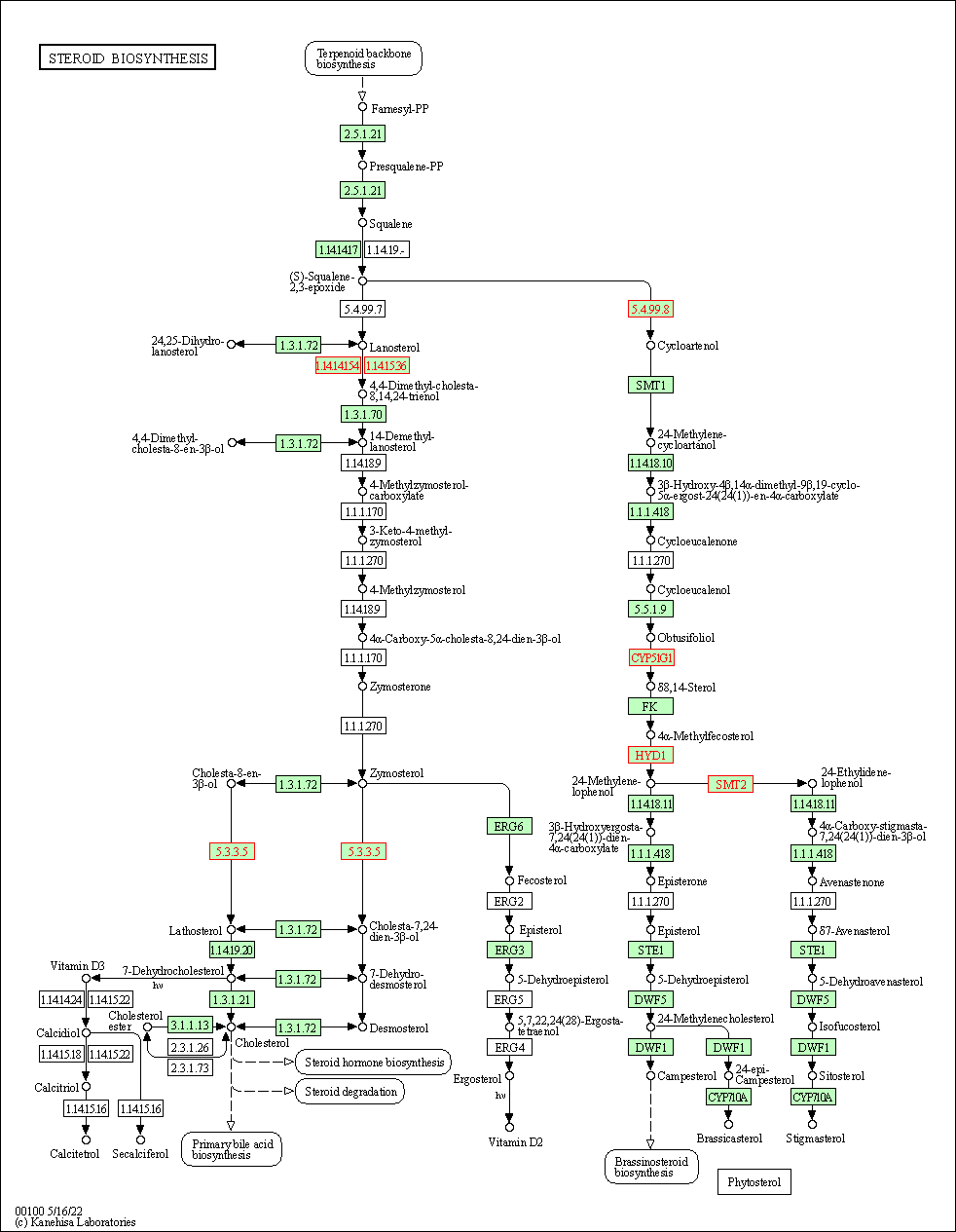 | 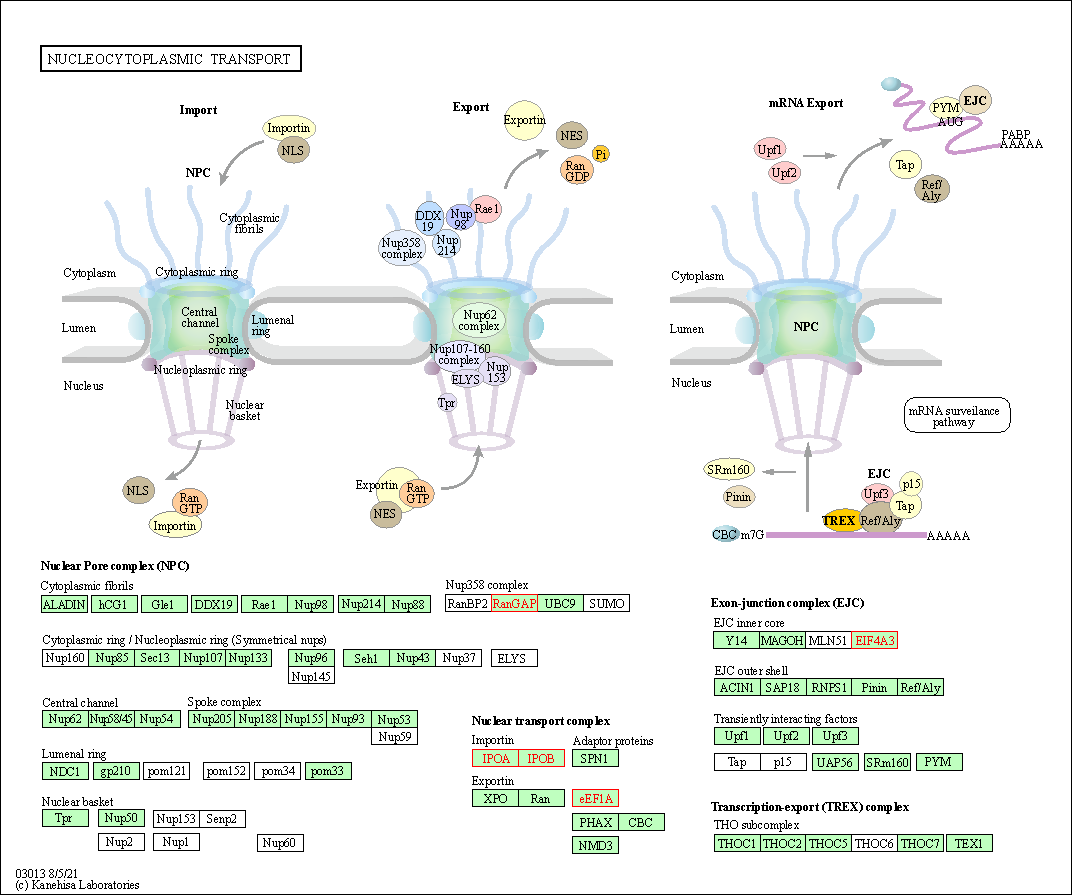 |
